# Molecular insights into ligand recognition and activation of chemokine receptors CCR2 and CCR3

**DOI:** 10.1038/s41421-022-00403-4

**Published:** 2022-05-15

**Authors:** Zhehua Shao, Yangxia Tan, Qingya Shen, Li Hou, Bingpeng Yao, Jiao Qin, Peiyu Xu, Chunyou Mao, Li-Nan Chen, Huibing Zhang, Dan-Dan Shen, Chao Zhang, Weijie Li, Xufei Du, Fei Li, Zhi-Hua Chen, Yi Jiang, H. Eric Xu, Songmin Ying, Honglei Ma, Yan Zhang, Huahao Shen

**Affiliations:** 1 Key Laboratory of Respiratory Disease of Zhejiang Province, Department of Respiratory and Critical Care Medicine, Second Affiliated Hospital of Zhejiang University School of Medicine, Hangzhou, Zhejiang China; 2 The CAS Key Laboratory of Receptor Research, Shanghai Institute of Materia Medica, Chinese Academy of Sciences, Shanghai, China; 3 School of Life Science and Technology, ShanghaiTech University, Shanghai, China; 4 University of Chinese Academy of Sciences, Beijing, China; 5 Department of Biophysics and Department of Pathology of Sir Run Run Shaw Hospital, Zhejiang University School of Medicine, Hangzhou, Zhejiang China; 6 Liangzhu Laboratory, Zhejiang University Medical Center, Hangzhou, Zhejiang China; 7 Department of Pharmacology and Department of Respiratory and Critical Care Medicine of the Second Affiliated Hospital, Zhejiang University School of Medicine, Key Laboratory of Respiratory Disease of Zhejiang Province, Hangzhou, Zhejiang China; 8 Department of Anatomy, Zhejiang University School of Medicine, Hangzhou, Zhejiang China; 9 International Institutes of Medicine, The Fourth Affiliated Hospital of Zhejiang University School of Medicine, Yiwu, Zhejiang China; 10 State Key Laboratory of Microbial Technology, Shandong University, Qingdao, Shandong China; 11 Zhejiang Provincial Key Laboratory of Immunity and Inflammatory Diseases, Hangzhou, Zhejiang China; 12 MOE Frontier Science Center for Brain Research and Brain-Machine Integration, Zhejiang University School of Medicine, Hangzhou, Zhejiang China; 13 State Key Lab of Respiratory Disease, Guangzhou, Guangdong China

**Keywords:** Cryoelectron microscopy, Chemotaxis

## Abstract

Chemokine receptors are a family of G-protein-coupled receptors with key roles in leukocyte migration and inflammatory responses. Here, we present cryo-electron microscopy structures of two human CC chemokine receptor–G-protein complexes: CCR2 bound to its endogenous ligand CCL2, and CCR3 in the apo state. The structure of the CCL2–CCR2–G-protein complex reveals that CCL2 inserts deeply into the extracellular half of the transmembrane domain, and forms substantial interactions with the receptor through the most N-terminal glutamine. Extensive hydrophobic and polar interactions are present between both two chemokine receptors and the Gα-protein, contributing to the constitutive activity of these receptors. Notably, complemented with functional experiments, the interactions around intracellular loop 2 of the receptors are found to be conserved and play a more critical role in G-protein activation than those around intracellular loop 3. Together, our findings provide structural insights into chemokine recognition and receptor activation, shedding lights on drug design targeting chemokine receptors.

## Introduction

The interactions of chemokines and their receptors are critical in inflammatory responses, which are associated with activation, differentiation, and migration of immune cells^1–5^. Chemokine receptors belong to class A G-protein-coupled receptors (GPCRs), which produce intracellular signaling pathways by recruiting heterotrimeric G-proteins^6^. To date, at least 20 chemokine receptors have been identified in human and are divided into various subfamilies according to the chemokine groups that they preferentially bind: CXC chemokine receptors, CC chemokine receptors, CX3C chemokine receptors, and XC chemokine receptors. Among them, the CC chemokine receptors form the largest subfamily of chemokine receptors and exhibit bidirectional promiscuity with their ligands: some chemokines can activate more than one subtypes of chemokine receptors, and many chemokine receptors can be activated by more than one chemokine^7,8^. Although significant progress has been made in structural studies of chemokine receptors in their active states, the molecular mechanisms underlying the activation and ligand recognition of CC chemokine receptors remain elusive^9–13^.

C–C chemokine receptor type 2 (CCR2) exhibits high ligand promiscuity among CC chemokine receptor family. At least four human CC chemokines, including CCL2, CCL7, CCL8, and CCL13 are identified as cognate ligands of CCR2^2^. Among them, CCL2 is the most potent activator of CCR2 signaling, and the upregulation of CCR2 by CCL2 is associated with cancer metastasis, relapse, and inflammatory diseases in the central nervous system, including multiple sclerosis, Alzheimer’s disease and ischemic stroke^14,15^. C–C chemokine receptor type 3 (CCR3) also belongs to the CC chemokine receptor family. The activation of CCR3 is associated with chemotaxis of eosinophils, thus considered as a well-established target for allergic diseases, such as asthma^16–18^. Activation of GPCRs stimulates the exchange of GTP for GDP in the Gα subunit^19,20^. The expression of CCR2 or CCR3 is sufficient to enable the nucleotide exchange, illustrating their high basal activity^21,22^. However, despite extensive research and pharmaceutical industry investment, no therapeutic small molecules targeting any of these receptors have been approved for clinical use^23,24^.

So far, only a few three-dimensional (3D) structures of CC chemokine receptors with their endogenous ligands have been determined: CCR1 in complex with CCL15; CCR5 in complex with CCL3 or CCL5; and CCR6 in complex with CCL20^11–13^. Although the receptors mentioned above belong to the same subfamily of chemokine receptors, the molecular mechanisms underlying the chemokine recognition, constitutive, and ligand-induced activation seems variable, which forms a complex regulatory network for the fine-tuning of chemokine-induced physiological responses. In this study, we employed single-particle cryo-electron microscopy (cryo-EM) to determine the structures of human CCR2 and CCR3 in complex with heterotrimeric G_i_ protein: CCR2 bound to its endogenous ligand CCL2, and CCR3 in the absence of ligand (apo) state, at resolutions of 2.9 Å and 3.1 Å, respectively. Together with mutagenesis and functional results, our studies reveal the structural basis of ligand recognition, receptor activation, and G_i_ protein coupling of these receptors. The structural information also provides multiple templates for rational design of novel therapeutics targeting CC chemokine receptors.

## Results

### Structural determination of CCL2–CCR2–G_i_ and apo CCR3–G_i_ complexes

To investigate the molecular mechanisms of chemokine receptors in ligand recognition and signal transduction, two endogenous ligands, CCL2 and CCL11, were used to form G-protein-coupled CCR2 and CCR3 complexes. To obtain a stable CCL2–CCR2–G_i_ complex, the wild-type full-length human CCR2 was modified by replacing the native signal peptide with prolactin precursor (PP), followed by a CCL2(1–72) sequence. A LgBiT and double maltose-binding protein (MBP) tag were inserted at the C-terminus to increase the receptor expression and enable the complex purification (Supplementary Fig. S1a, b)^25–27^. The CCL2–CCR2 chimera and the heterotrimeric G_i_ protein were co-expressed in insect cells. The CCL2–CCR2–G_i_ complex was assembled on the membrane by incubation with apyrase and the Gα- and Gβ-binding antibody scFv16.

**Fig. 1:**
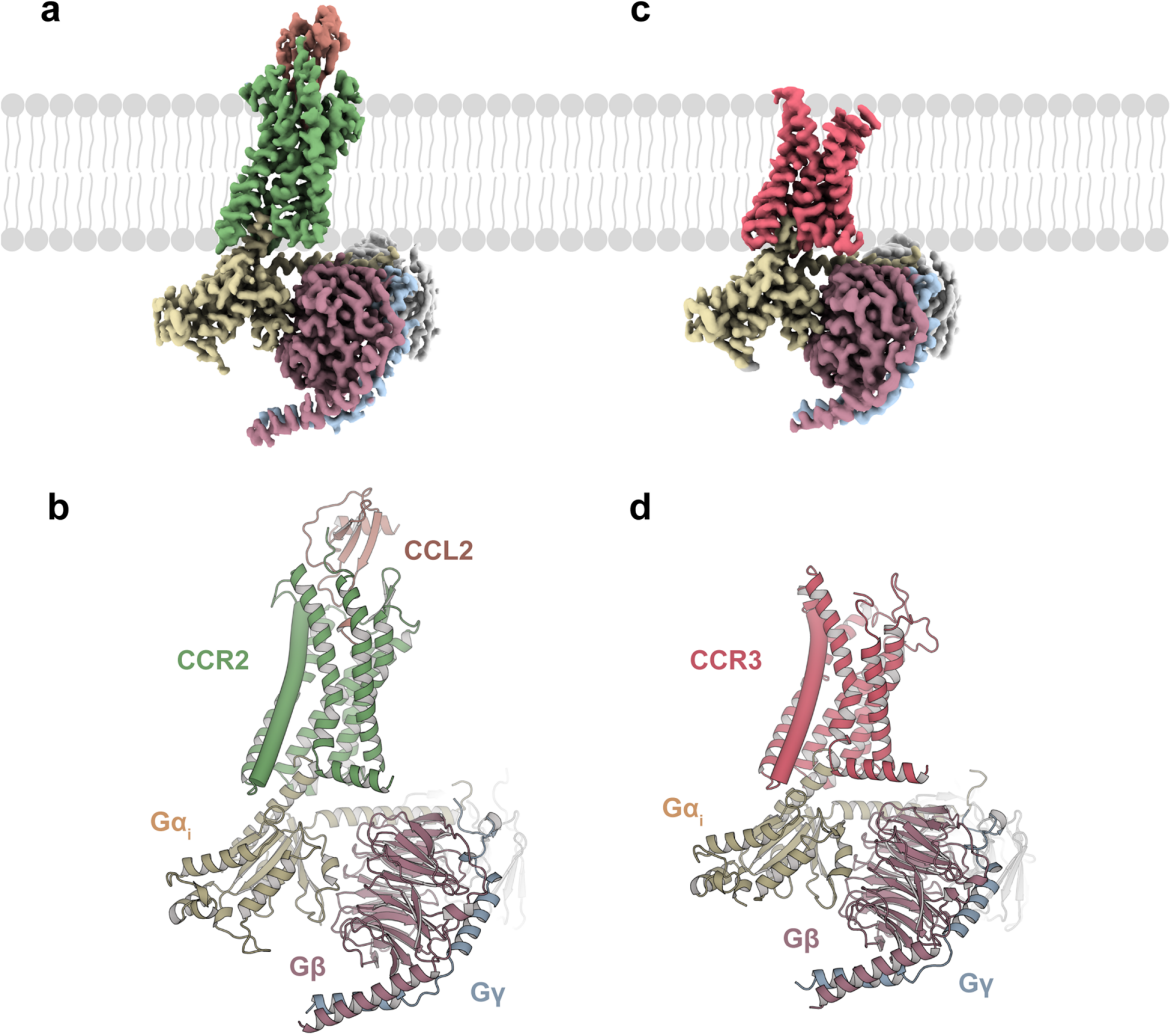
Cryo-EM structures of the CCL2–CCR2–G_i_ and apo CCR3–G_i_ complexes. **a**, **c** Cryo-EM density maps of the CCL2–CCR2–G_i_ (**a**) and apo CCR3–G_i_ complexes (**c**). **b**, **d** Models of the CCL2–CCR2–G_i_ (**b**) and apo CCR3–G_i_ complexes (**d**). Rosy brown, Gβ; light blue, Gγ; gray, scFv16; green, CCR2 (**a**, **b**); pink, CCR3 (**c**, **d**); yellow, Gα_i1_; brown, CCL2 (**a**, **b**).

Following a similar strategy, we sought to obtain the CCL11–CCR3–G_i_ complex. A single-point mutation (I244^6.40^A) of CCR3 was introduced to increase the constitutive activity of the receptor (superscripts indicate the Ballesteros–Weinstein numbering scheme) (Supplementary Fig. S1a, c, d)^28^. Using single-particle cryo-EM, the structure of CCL2–CCR2–G_i_ was determined with an overall resolution of 2.9 Å (Fig. 1a, b; Supplementary Fig. S2a, b). However, CCL11 was absent from the orthosteric pocket of CCR3, and the structure of the apo CCR3–G_i_ complex was determined with an overall resolution of 3.1 Å (Fig. 1c, d; Supplementary Fig. S2c, d). The EM density maps were well resolved, enabling unambiguous placement of the receptor (residues 29–323 for CCL2-bound CCR2 and residues 57–387 for the apo CCR3), the G_i_ protein heterotrimer, scFv16 and CCL2 in the complexes (Supplementary Fig. S3 and Table S1).

### Ligand recognition of chemokine receptors

**Fig. 2:**
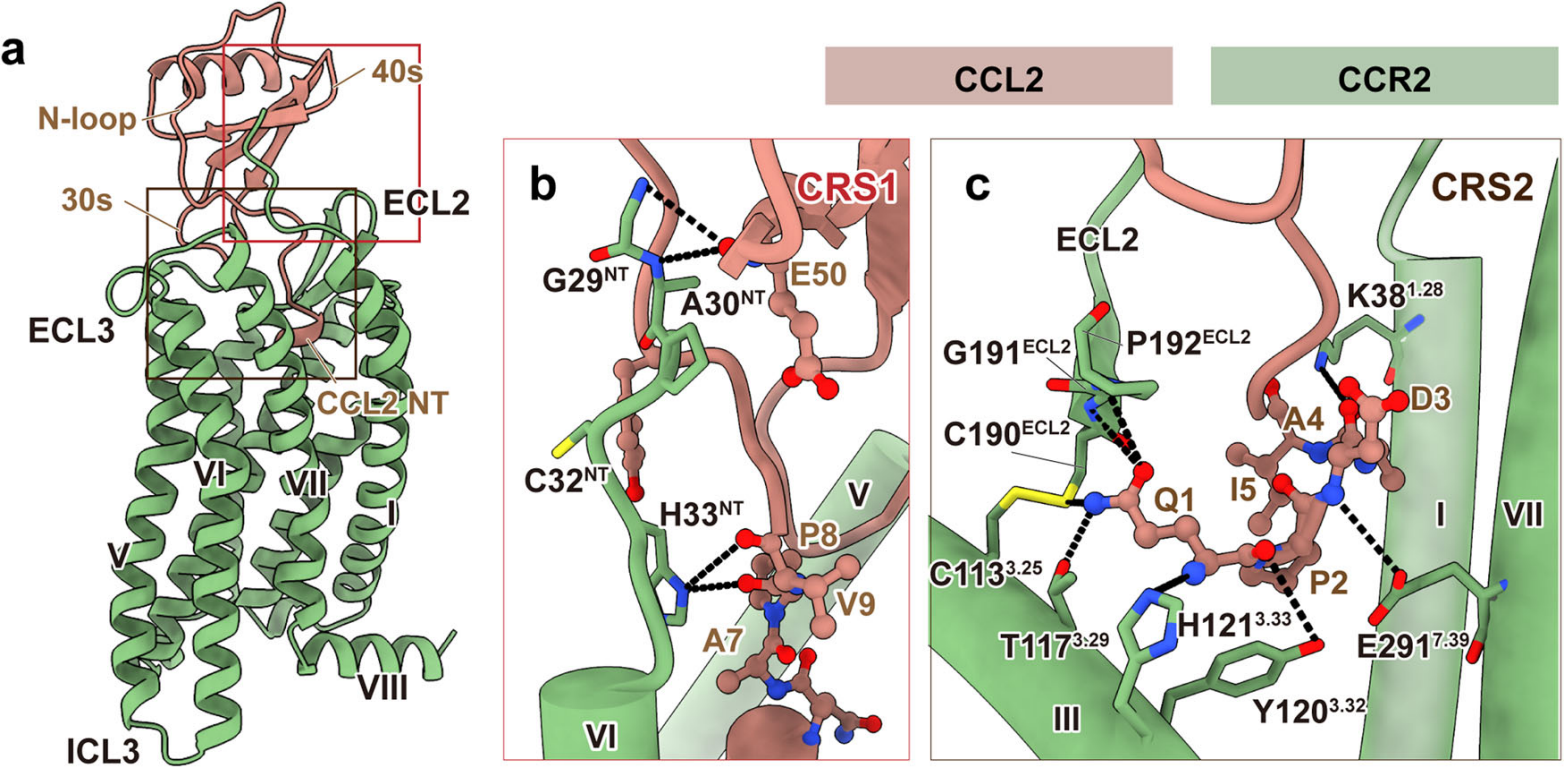
The orthosteric chemokine-binding pocket of CCR2. **a** Side view of CCR2 (green) bound to its endogenous ligand CCL2 (brown). **b**, **c** Details of interactions between CCR2 and CCL2 at the CRS1 (**b**) and CRS2 (**c**) regions. Hydrogen bonds are depicted as black dashed lines.

The CCL2-bound CCR2 structure revealed that CCL2 was stably anchored in the extracellular half of the receptor transmembrane domain (TMD) and formed extensive interactions with the N-terminus, ECL2, and TM helices (except TM4) of CCR2 (Fig. 2a). The ligand recognition mode observed in the CCL2–CCR2–G_i_ complex was consistent with the classic “two-site” model, which involves two main interaction sites between chemokines and their receptors: (1) chemokine recognition site 1 (CRS1) is at the N-terminus of the receptor, which interacts with the globular cores of chemokines; (2) chemokine recognition site 2 (CRS2) is within the TMD pocket of receptors, where the N-termini of chemokines are bound^29,30^. At the CRS1, the N-terminus of CCR2 (residues ^29^GAPCH^33^) formed several hydrophobic and hydrogen bonds with the N-terminus, N-loop, and nearby β3 region of CCL2 (Fig. 2b). In agreement with that, C32^NT^A substitution significantly impaired the activation of the receptor (Supplementary Fig. S4a). By contrast, the EM map of apo CCR3 showed no density at the N-terminus, suggesting that the interactions with chemokines contributed to stabilizing the N-terminal region of the receptors (Supplementary Fig. S3d).

At CRS2, CCL2 was observed to locate at the orthosteric binding pockets of the receptor, which were conventionally divided into a minor pocket (composed of TM1–3 and TM7) and a major pocket (composed of TM3–7)^31^. Consistent with other endogenous ligands, the N-terminus of CCL2 faced towards the minor pocket of CCR2. The insertion depth of CCL2 was similar to those observed in the CCL15–CCR1 (PDB: 7VL9), CCL3–CCR5 (PDB: 7F1Q), and CCL5–CCR5 (PDB: 7F1R) complexes, but contrasted markedly with the shallow binding mode observed in the CCL20–CCR6 complex (PDB: 6WWZ) (Supplementary Fig. S4b). In detail, the N-terminus of CCL2 (residues ^1^QPDAINAPVT^10^) was deeply inserted into the extracellular pocket of TMD. The most N-terminal glutamine of CCL2 directly interacted with CCR2, forming extensive hydrogen bonds with the extracellular end of TM3 (C113^3.25^, T117^3.29^, Y120^3.32^, and H121^3.33^) and ECL2 (C190^ECL2^, G191^ECL2^, and P192^ECL2^) (Fig. 2c; Supplementary Table S2). Sequence alignment showed that the most N-terminal glutamine and the insertion length of the N-terminus were both identical among endogenous agonists of CCR2 (Supplementary Fig. S4c). These results suggested that the most N-terminal glutamine present in chemokines had an important role in the recognition specificity of CCR2. Furthermore, the mutation of either C190^ECL2^ or Y120^3.32^ to alanine on CCR2 significantly impaired the G-protein activation of the receptor, indicating that these residues played a pivotal role in the agonism of CCL2 (Supplementary Fig. S4a).

Besides, the overall pose of chemokine relative to CCR2 is remarkably similar to that in CCR6–G but different from those in CCR1–G and CCR5–G complexes. As shown in Supplementary Fig. S5, the overall pose of CCL2 to CCR2 was similar to that of CCL20 to CCR6, but rotated by ~50° when compared to the corresponding chemokines in the CCL15–CCR1 and CCL3/CCL5–CCR5 complexes, suggesting diverse recognition modes among these chemokine–receptor interactions.

### Activation of CCR2 and CCR3

**Fig. 3:**
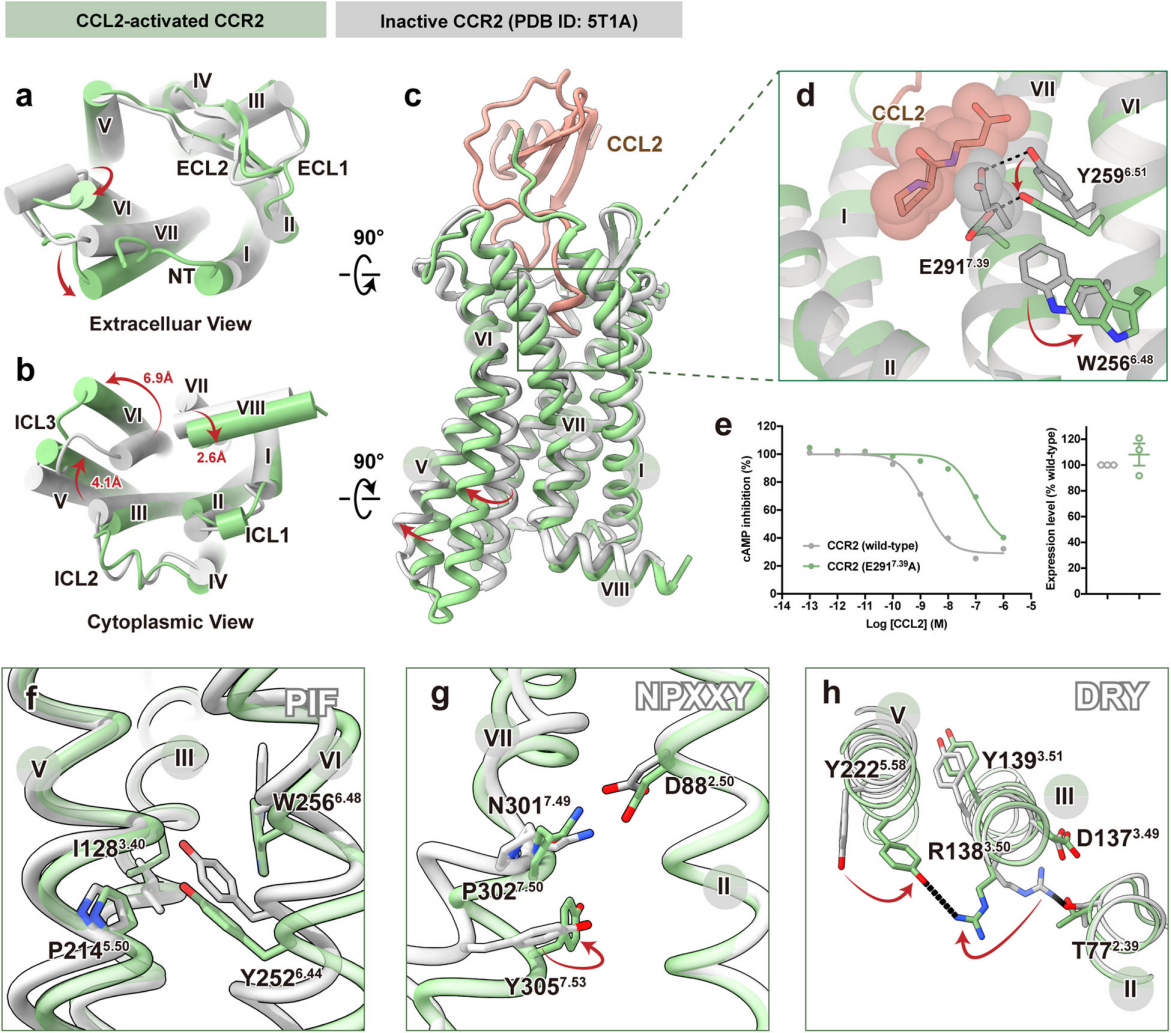
Molecular basis for activation and G-protein coupling of CCR2 induced by CCL2. **a**–**c** Extracellular (**a**), cytoplasmic (**b**), and side view (**c**) of CCL2-bound CCR2 (green, active state) superimposed on the inactive CCR2 (gray, PDB: 5T1A). **d** Close-up view of the orthosteric binding pocket in CCR2. The N-terminal residues of CCL2 (brown) and E291^7.39^ (gray) of inactive CCR2 are shown as spheres. **e** Dose-response curves for CCL2-induced G_i_ signaling on CCR2 (wild-type) and CCR2 (E291^7.39^A) measured by Glosensor assay. Data are shown as means ± SEM; *n* = 3 independent experiments, performed with single replicates. **f**–**h** Close-up views of the conserved PIF (**f**), NPxxY (**g**), and DRY (**h**) motifs showing conformational changes along the pathway of receptor activation. Hydrogen bonds are depicted as black dashed lines.

Structural comparison between the receptors in the CCL2–CCR2–G_i_ complex and inactive CCR2 (PDB: 5T1A) was performed to determine the molecular mechanism of CCR2 activation^32^. On the extracellular side, CCL2 induced significant outward movement of TM6 and inward movement of TM7 (Fig. 3a). Substantial structural changes were observed on the cytoplasmic side, in particular, rearrangements of TM5–7 and H8. Major changes occurred in TM6, which was rotated outward from the center of the TM bundle with a distance of 6.9 Å (with A^6.33^ as a reference) in CCR2 to accommodate the α5-helix of Gα_i_. The conformational change was also accompanied by the movement of TM5 towards TM6 by ~4.1 Å (with L^5.66^ as a reference), along with the shift of H8 towards TM1 by ~2.6 Å (with F^8.50^ as a reference) (Fig. 3b).

With the insertion of CCL2 in the orthosteric pocket of CCR2, the distal N-terminus of the ligand pushed residue E291^7.39^ of CCR2 downwards to the cytoplasmic side. Y259^6.51^ was sandwiched between E291^7.39^ and W256^6.48^. As a result, the movement of E291^7.39^ led to a deflection of Y259^6.51^, and then induced the side-chain rearrangement of W256^6.48^ by steric hindrance. The highly conserved residue W^6.48^ has been reported to function as a “toggle switch” in most class A receptors, triggering the activation motion of GPCRs (Fig. 3c, d). Consistent with that, E291^7.39^A substitution significantly impaired the ligand-induced receptor activation by 63 folds (Fig. 3e). In this complex, the resulting movement of W256^6.48^ further gave rise to rearrangements of the conserved PIF motif (P214^5.50^, I128^3.40^, and Y252^6.44^ in CCR2), which led to outward movement of TM6 (Fig. 3f). Subsequently, the activation signal was propagated through the conserved NPxxY motif (N301^7.49^, P302^7.50^, and Y305^7.53^ in CCR2) to the bottom DRY motif (D137^3.49^, R138^3.50^, and Y139^3.51^ in CCR2) (Fig. 3g, h), which ultimately led to the outward movement of TM6 to accommodate the binding of G-proteins.

Despite of the absence of ligand, the conformation of apo CCR3 was similar to that of CCR2 activated by CCL2, except that the extracellular TM5 of CCR3 moved outward towards TM6 by ~3.6 Å (with W^5.34^ as a reference). The intracellular end of TM6 of CCR3 was rotated inward by 2.5 Å (with A^6.33^ as a reference) compared with that of CCR2 (Supplementary Fig. S6a–c). Conformations of the residues involved in conserved microswitches were similar between these two receptors, suggesting a shared activation mechanism (Supplementary Fig. S6d–f). Interestingly, the conserved “ionic lock” in the inactive state (the salt bridge between R^3.50^ of the DRY motif and D/E^6.30^ of GPCRs) appeared to be defective, owing to the replacement of D/E^6.30^ by lysine or arginine in chemokine receptors. R^3.50^ of the DRY motif in both CCL2-bound CCR2 and apo CCR3 formed a hydrogen bond with Y^5.58^ instead, a conserved residue among all chemokine receptors, sustaining the active state (Fig. 3h; Supplementary Fig. S6f).

### Interfaces between chemokine receptors and Gα_i_

Among all published structures of CC chemokine receptors in complex with endogenous ligands, CCR1 showed high intrinsic constitutive activity and sequence similarity with both CCR2 and CCR3 (approximate similarity of 72.4% and 83.6%, respectively). To probe the molecular mechanism of G-protein coupling by CC chemokine receptors, we compared the receptor–G_i_ interface among the complexes of CCL15–CCR1–G_i_ (PDB ID: 7VL9^13^), CCL2–CCR2–G_i_, and apo CCR3–G_i_. Globally, these three complexes were similar in receptor–Gα_i_ protein interaction with other class A GPCRs: the α5-helix of Gα_i_ inserted into the cytoplasmic core of TMD, forming extensive hydrophobic interactions with the TM3, intracellular loop 2 (ICL2), TM5, ICL3 and TM6 of the receptor (Supplementary Fig. S7a)^10,33–35^. Most of the interactions were concentrated in the two regions: (1) ICL2 and the intracellular end of TM3; (2) ICL3 and the intracellular ends of TM5 and TM6. Interestingly, structural alignments revealed that the interactions around ICL2 seemed to be relatively conserved among these three complexes, but differed in the region around ICL3 (Supplementary Fig. S7b).

**Fig. 4:**
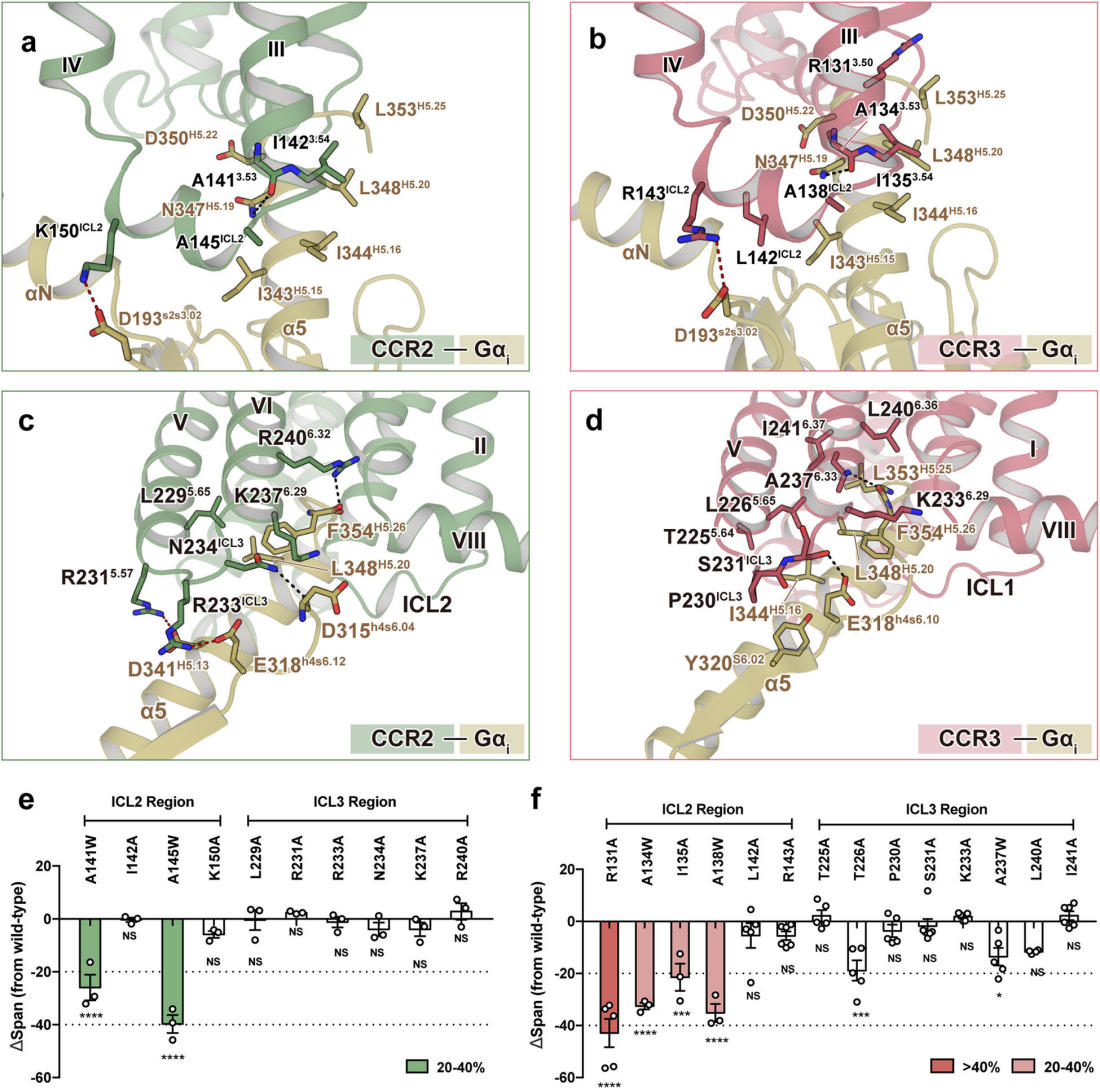
Interactions between chemokine receptors and Gα_i1_. **a**, **b** Magnified view of the interactions between TM3 and ICL2 of CCR2 (**a**) and CCR3 (**b**) with the α5-helix of Gα_i_. **c**, **d** Detailed interactions of TM5, TM6, and ICL3 of CCR2 (**c**) and CCR3 (**d**) with the α5-helix of Gα_i_. Green, CCL2-bound CCR2; pink, CCR3; yellow, Gα_i1_. Hydrogen bonds are depicted as black dashed lines, and electrostatic bonds are depicted as red dashed lines. **e**, **f** Influence of single-site mutants of CCR2 (**e**) and CCR3 (**f**) on agonist-induced cAMP accumulation. *n* = at least three independent experiments performed with single replicates. The statistical difference between wild-type and mutated receptor was calculated by one-way ANOVA followed by Dunnett’s multiple comparisons test. Superscripts indicate statistically significant difference (*P < 0.01, ***P < 0.001, ****P < 0.0001; NS no significance). Bars are colored according to the extent of effect. All data are shown as means ± SEM. See Supplementary Tables S5 and S6 for detailed statistical evaluation.

In the CCL2–CCR2–G_i_ complex, a vast number of residues located around ICL2 directly interacted with Gα_i_; these interactions included electrostatic or hydrogen bonds formed by A141^3.53^ and K150^ICL2^, as well as hydrophobic interactions formed by I142^3.54^ and A145^ICL2^ with Gα_i_ (Fig. 4a; Supplementary Table S3). Similar interactions were also observed in the complexes of activated CCR1 and CCR3 (Fig. 4b; Supplementary Fig. S7c). In contrast, the G-protein interface around ICL3 differed among these three complexes: two hydrogen bond interactions (R240^6.32^:F354^H5.26^ and N234^ICL3^:D315^h4s6.04^) and an electrostatic bond interaction (R231^5.57^:D341^H5.13^) were observed in the interface between CCL2-bound CCR2 and Gα_i_ (the superscripts following the Gα residues are based on the common Gα numbering system) (Fig. 4c; Supplementary Table S3)^36^; more interactions were observed in the apo CCR3–G_i_ complex, including two hydrogen bond interactions (A237^6.33^:L353^H5.25^ and S231^ICL3^:E318^h4s6.10^), and many hydrophobic interactions formed by T225^5.64^, L226^5.65^, P230^ICL3^, K233^6.29^, L240^6.36^ and I241^6.37^ of CCR3 with Gα_i_ (Fig. 4d; Supplementary Table S4). Besides, in the region around ICL3 of CCR1, only one electrostatic bond formed by R229^5.68^ was found (Fig. 4b; Supplementary Fig. S7d). Therefore, we speculated that the ICL2–Gα_i_ interface was conserved in chemokine receptors, which could play a more important role in G-protein-coupling than the ICL3–Gα_i_ interface.

Consistently, most of single-point mutations in the ICL2 region of CCR2 and CCR3 significantly decreased the agonist-induced activation (Fig. 4e, f; Supplementary Tables S5, S6). Alanine substitutions in the residues around ICL2 (R131^3.50^, L142^ICL2^, and R143^ICL2^) were observed to reduce the constitutive activity of CCR3 (Supplementary Fig. S8a–c). However, mutations in the ICL3 region of either CCR2 or CCR3 had a smaller effect (Fig. 4e, f; Supplementary Tables S5, S6). Notably, although several single-point mutations of CCR1 (R131^3.50^ and A138^ICL2^) exhibited reduced constitutive activity, most single-point mutations of CCR1 in the ICL2 region (R131^3.50^A, A134^3.53^W, I135^3.54^A, A138^ICL2^W, and L142^ICL2^A) had little influence on the agonist-induced activity of the receptor (Supplementary Fig. S8d, e and Table S7). However, multiple point mutations of residues around ICL2 nearly abolished the agonist-induced activation and significantly decreased the constitutive activity of CCR1, suggesting that the interactions in the ICL2–Gα region were invulnerable to residue substitution during evolution to maintain G-protein-coupling activity (Supplementary Fig. S8f, g).

## Discussion

In this work, we reported the cryo-EM structures of CCR2 and CCR3 in complex with heterotrimeric G_i_ protein. The structure of the CCL2–CCR2–G_i_ complex revealed the contribution of the most N-terminal glutamine to ligand recognition and receptor activation, which is conserved in almost all chemokines targeting CCR2. Moreover, both CCL2-bound CCR2 and apo CCR3 form strong interactions with Gα_i_, suggesting a common mechanism involved in the constitutive activity of CC chemokine receptors.

Structural composition among CCL2–CCR2 and the other available CC chemokine–receptor complexes revealed two distinct ligand recognition modes amongst CC chemokine–CC receptor system: (1) the binding mode observed in CCL2–CCR2 and CCL20–CCR6 complexes, highly consistent with the classical “two-site” model; (2) the binding mode observed in CCL15–CCR1 and CCL3/5–CCR5 complexes, in which additional interactions beyond the “two-site” model are involved. In the former, the buried surface area of CRS2, which is formed by receptors and the N-terminus of ligands, accounts for a larger proportion of the whole interactions. The most N-terminal residues of CCL2 and CCL20 both function as a determinant in the chemokine recognition and activation of their receptors^11^. However, the ligands of CCR1 and CCR5 display diversity in the N-terminal segment proceeding the first two cysteines, but exhibited evident similarity within the core region^12,13^.

Structural information of CCR1, CCR2, and CCR3 in complex with heterotrimeric G-proteins indicates that the residues at ICL2 region, rather than those located around ICL3, form more conserved interactions with the α5-helix of Gα_i_. Consistently, functional experiments revealed significant loss of G-protein activation of these three CC chemokine receptors by point mutations of residues around ICL2 region, whereas mutations in ICL3 only had minor effects. Therefore, we propose that the ICL2 region is indispensable in complex formation of receptor–G-protein, thus contributing to G-protein activation. Consistently, recent studies determined that positive allosteric modulators could achieve their efficacy by stabilizing ICL2 in the active conformation, illustrating the significance of ICL2 region in G-protein coupling^37,38^.

Taken together, the cryo-EM structures we report here provide insights into ligand recognition and direct structural evidence for the G-protein activation by CCR2 and CCR3. Our study also complements the molecular mechanisms of immunomodulation mediated by chemokine receptors, which will allow rational design of more selective drugs targeting these receptors.

## Materials and methods

### Expression and purification of chemokines

In this study, CCL2(1–72), CCL15(26–92), and CCL24(1–93) were employed in functional experiments as an agonist targeting CCR2 and CCR3, respectively. For the purification of CCL2(1–72), the sequence of CCL2(1–72) was cloned into a modified pFastBac1 vector, which contained a GP67 signal peptide at the N-terminus prior to the ligand to facilitate protein secretion. An MBP-tag followed by a C-terminal 8× His-tag was fused into the C-terminus of CCL2(1–72) with a linker containing a 3C protease cleavage site (LEVLFQGP) for further purification. Using a Bac-to-Bac system, CCL2(1–72)-3C-MBP-8× His was overexpressed in High Five insect cells. Cell cultures were grown in protein-free insect cell culture medium (Expression Systems ESF 921). After expression for 48 h, the culture medium of the infected cells was collected and initial purification was performed using Ni-NTA affinity chromatography (GE Healthcare). CCL2(1–72)-3C-MBP-8× His was eluted with high-imidazole elution buffer (20 mM HEPES, pH 7.5, 100 mM NaCl, and 250 mM imidazole). Then, the C-terminal MBP and 8× His-tag were removed by 3C protease digestion; 10% (w/v) glycerol was added together with 3C protease to decrease protein precipitation. Finally, purification to homogeneity of CCL2(1–72) was achieved by size-exclusion chromatography on a Superdex^TM^ 75 Increase 10/300 GL column (GE Healthcare) in SEC buffer (20 mM HEPES, pH 7.5, 100 mM NaCl, and 10% (w/v) glycerol); this also resulted in the separation of CCL15(30–113), 3C protease, and the MPB fusion protein. CCL15(26–92) and CCL24(1–93) were obtained by following a similar strategy as described above, with the sequence of CCL2(1–72) replaced by the sequence of CCL15(26–92) and CCL24(1–93) in the construction of recombinant plasmids, respectively.

### Purification of scFv16

The expression and purification of scFv16 were performed as previously described^39^. In brief, scFv16 secretion in the supernatant of transfected High Five cells was collected by affinity chromatography using Ni-Sepharose columns. The eluate was purified by size-exclusion chromatography with a Superdex^TM^ 200 Increase 10/300 GL column (GE Healthcare). Then, the monomeric fractions were concentrated, flash frozen, and stored at −80 °C until use.

### cAMP inhibition assay (GloSensor)

In this study, the dose-response curve of cAMP accumulation was performed as previously described^13^. For the measurement of constitutive activity, we fused a FLAG-tag into the N-terminus of receptors, and cloned it into pcDNA3.1 plasmids. HEK 293 T cells were transfected with a plasmid mixture consisting of pcDNA3.1-Flag-GPCR and the cAMP biosensor GloSensor-22F (Promega) at a ratio of 3:1. The cells transfected with GloSensor-22F and the empty pcDNA3.1 plasmid were served as a blank control. After 24 h, transfected cells were plated on a 96-well plate coated with polylysine (Applygen, #C1010). After 12 h, cells were treated with Hanks’ Balanced Salt Solution (HBSS) for starvation and then incubated in CO_2_-independent medium containing 2% GloSensor cAMP Reagent (Promega) at a volume of 50 μL per well. After 30 min of incubation at room temperature, the baseline luminescence was measured using a Spark Multimode microplate reader (TECAN); 1 μM Forskolin (5.5 μL) (Sigma) was added to each well before the second luminescence reading. Luminescence counts were first normalized to the initial count. The difference of fold-change signals between cells transfected with the empty pcDNA3.1 plasmid (ΔLum_blank) and those transfected with the pcDNA3.1 plasmid containing the sequence of wild-type GPCR (ΔLum_WT) reflected the constitutive activity of wild-type GPCR, which was used as a reference value set to 100%. The calculation formula of the constitutive activity is: Constitutive Activity (%) = (ΔLum_blank – ΔLum_Mut)/ (ΔLum_blank – ΔLum_WT) × 100%.

### NanoBiT G-protein dissociation assay

The NanoBiT G-protein dissociation assay was performed for the measurement of G-protein activation as previously described. A large fragment (LgBiT) of the NanoBiT luciferase was inserted into the helical domain (between the αA and the αB helices) of a Gα subunit. A small fragment (SmBiT) was N-terminally fused to a C68S-mutated Gγ2 subunit. HEK 293 T cells were co-transfected with a plasmid mixture consisting of pcDNA3.1-LgBiT-Gα_i_, pcDNA3.1-Gβ1, pcDNA3.1-SmBiT-Gγ2, and pcDNA3.1-CCR1 in a 1:5:5:2 ratio. After 1 day of incubation, cells were plated onto a 384-well plate coated with polylysine (Applygen, #C1010). After 12 h, cells were washed three times with 0.5 mM EDTA-containing Dulbecco’s phosphate-buffered saline (DPBS) to remove cell medium and loaded with 25 μL of 5 μM coelenterazine h (Yeasen) diluted in assay buffer (HBSS containing 0.01% bovine serum albumin and 5 mM HEPES, pH 7.5) per well. After 2 h of incubation at room temperature, the plate was measured for baseline luminescence (TECAN). Then, the ligand was added at different concentrations before taking the second luminescence measurement. Luminescence counts were first normalized to the initial count, and then fold-changes in signals compared with those obtained with the lowest ligand concentration were set as 100% in each experiment. Nonlinear regression analysis was performed using a sigmoidal dose-response in GraphPad Prism to calculate the values of *E*_max_ and EC_50_.

### Expression and purification of CCL2–CCR2–G_i_ and apo CCR3–G_i_ complexes

In this work, a NanoBiT tethering strategy was employed for complex stabilization^25–27^. For the purification of CCL2–CCR2–G_i_, the sequence of human CCR2 (residues 1–355) was fused with a LgBiT subunit (Promega) at the C-terminus followed by a double MBP-tag via a GS linker containing a TEV protease cleavage site (ENLYFQG). The sequence of CCL2(1–72) was fused to the N-terminus of CCR2. For the expression of the apo CCR3–G_i_ complex, a constitutive active mutation was introduced (I244^6.40^A). CCR3 was fused with a 6× His-tag at the N-terminus and a following BRIL (MBP) fusion protein via a linker containing a TEV protease site (ENLYFQG). And the sequence of prolactin precursor (PP) signal peptide was fused into the N-terminus of CCL3(4–69) and BRIL-CCR3. In this study, the human CCL11(1–74) was also purified for use in the formation of the CCR3–G_i_ complex. First, we cloned the cDNA sequence of CCL11(1–74) into a pETDuet vector with an N-terminal SUMO and 6× His-tag. The CCL11 expression vector was transformed into *Escherichia coli* BL21(DE3) cells. After overnight expression at 16 °C, an Ni-chelating HP Sepharose column (GE Healthcare) was used for purification of 6× His-SUMO-CCL11. Then, the purified protein was desalted and treated with ULP1 protease to remove the 6× His-SUMO-tag. The cleaved protein was passed through the Ni-chelating HP Sepharose column again, and CCL11 without the tag was collected as flow-through. Finally, CCL11 was purified by gel filtration chromatography with a HiLoad 26/60 Superdex 200 column (GE Healthcare) in 20 mM Tris, pH 8.0, 150 mM NaCl, and 10% glycerol.

The sf9 insect cells were infected with viruses encoding CCL2–CCR2–LgBiT/CCR3–LgBiT, Gα_i_, Gβ1–peptide86, Gγ2, and scFv16 at a ratio of 1.5:1:1:1:2. After 48 h expression, the infected cells were collected and resuspended in 20 mM HEPES, pH 7.5, 100 mM NaCl, 20 mM KCl, 10 mM MgCl_2_, and 5 mM CaCl_2_ supplemented with an EDTA-free protease inhibitor cocktail (TargetMol). Then, 10 μM of purified CCL11 was added for the activation of CCR3, and the membrane was solubilized with 0.5% (w/v) *n*-dodecyl β-d-maltoside (DDM, Anatrace), 0.01% (w/v) LMNG, and 0.1% (w/v) CHS for 2 h at 4 °C. For the CCL2–CCR2–G_i_ complex, the supernatant was collected and incubated with amylose resin (NEB). After binding, the resin was packed into a gravity-flow column and washed in 20 mM HEPES, pH 7.5, 100 mM NaCl, 2 mM MgCl_2_, 0.01% (w/v) LMNG, 0.01% (w/v) GDN (Anatrace), and 0.004% (w/v) CHS. TEV protease was then added to remove the C-terminal MBP, the cleaved protein was passed through Amylose resin again, and the complexes without MBP were collected as flow-through. For the purification of the CCR3–G_i_ complex, the supernatant was isolated and incubated at 4 °C with TALON^®^ Metal Affinity Resin. After binding, the talon resin with protein complex was loaded onto a gravity-flow column. The talon resin was washed with 20 column volumes of 20 mM HEPES, pH 7.5, 100 mM NaCl, 25 mM imidazole, 0.01% (w/v) LMNG, 0.005% (w/v) GDN (Anatrace), and 0.004% (w/v) CHS and eluted with the same buffer plus 300 mM imidazole. The complex was concentrated and loaded onto a Superdex 200 10/300 GL Increase column (GE Healthcare), and monomeric CCL2–CCR2–G_i_ and apo CCR3–G_i_ complexes were collected in 20 mM HEPES, 100 mM NaCl, pH 7.5, 2 mM MgCl_2_, 0.0005% (w/v) LMNG, 0.00025% GDN, and 0.0002% (w/v) CHS.

### Cryo-EM grid preparation and data collection

Three microliters of the purified CCL2–CCR2–G_i_ and apo CCR3–G_i_ complexes at ~5 mg/mL were applied to a glow-discharged holey carbon grid (Quantifoil R1.2/1.3). Grids were plunge-frozen in liquid ethane using Vitrobot Mark IV (Thermo Fischer Scientific). Frozen grids were transferred to liquid nitrogen and stored for data collection. For the CCL2–CCR2–G_i_ complex, cryo-EM imaging was performed on a Titan Krios at 300 kV using a Gatan K2 Summit detector at the Center of Cryo-Electron Microscopy, Zhejiang University (Hangzhou, China); 3190 movies of the CCL2–CCR2–G_i_ complex were recorded in counting mode at a dose rate of ~8.0 e^−^/Å^2^/s with a defocus ranging from −0.5 to −2.5 μm using the SerialEM software^40^. The total exposure time was 8 s, and 40 frames were recorded per micrograph. For the apo CCR3–G_i_ complex, cryo-EM imaging was performed on a Titan Krios at 300 kV using a Gatan K3 Summit detector at Shuimu BioSciences Ltd. (Beijing, China); 2695 movies were recorded in counting mode at a dose rate of ~18.0 e^−^/Å^2^/s with a defocus ranging from −1.2 to −2.2 μm. The total exposure time was 3.3 s, and 32 frames were recorded per micrograph.

### Image processing and map construction

Cryo-EM image stacks were aligned using MotionCor2.1^41^. Contrast transfer function (CTF) parameters were estimated by Gctf^42^. Particle selections for two-dimensional (2D) and 3D classifications were performed on a binned dataset using RELION-3.0-beta2^43^. For the CCL2–CCR2–G_i_ complex, 1,899,329 particles yielded by automated particle picking were subjected to 2D classification, and two rounds of 3D classification using the CCL15–CCR1–G_i_ complex low-pass filtered map as an initial reference model, resulting in a well-defined subset with 529,703 particles^13^. The selected subsets were subsequently subjected to 3D classification with a mask on the receptor or receptor–G_i_ complex, respectively. High-quality particles were selected from the intersection of the best classes from these two 3D classifications, producing 142,391 particles. The selected particles were subsequently subjected to 3D refinement, CTF refinement, and Bayesian polishing, generating a map with an indicated global resolution of 2.8 Å at a Fourier shell correlation of 0.143.

For the apo CCR3–G_i_ complex, 2,064,401 particles yielded by automated particle picking were subjected to 2D classification, and two rounds of 3D classification using the 5-HT_1E_ complex low-pass filtered map as an initial reference model, resulting in two well-defined subsets with 667,307 particles^34^. The selected subsets were subsequently subjected to a further round of 3D classification. High-quality particles were selected from the intersection of the good classes from these two 3D classifications, producing 373,892 particles. The selected particles were subsequently subjected to 3D refinement, CTF refinement, and Bayesian polishing, generating a map with an indicated global resolution of 3.1 Å at a Fourier shell correlation of 0.143.

### Model building and refinement

For the CCL2–CCR2–G_i_ complex, the initial model for CCR2 was the crystal structure of inactive CCR2 (PDB: 5T1A), and the initial model for CCL2 was the crystal structure of CCL2 (PDB: 1DOK) from the Protein Data Bank^32,44^. The initial model for the G_i_–scFv16 complex was generated from the μ opioid receptor–G_i_ complex (PDB: 6DDF)^45^. The models were docked into the cryo-EM density map using Chimera^46^. After the initial docked models had been refined using Rosetta, the models were subjected to iterative rounds of manual adjustment and auto refinement in Coot and Phenix, respectively^47,48^. The final refinement scores were validated by the module “comprehensive validation (cryo-EM)” in Phenix^48^. Structure figures were prepared using PyMOL (https://pymol.org/2/), Chimera, and ChimeraX^46,49^.

### Flow cytometric analysis of receptor expression

Flow cytometric analyses were performed using a CytoFlex (Beckman CytoFlex, USA). The transfected cells were stained with PE anti-FLAG (Biolegend). Cells were gated by FSC-A versus SSC-A to exclude debris and then by FSC-A versus FSC-H to exclude cell doublets. Mean PE fluorescence intensities were determined from over 5000 cells per experiment, which reflected the membrane protein expression level. All data for mutated receptors were normalized to the expression level of wild-type receptor in the same experiment. Values are shown as a percentage of the wild-type value, which was set to 100%.

## Supplementary information

Supplementary Information

## Data Availability

Cryo-EM maps of the CCL2–CCR2–G_i_ and apo CCR3–G_i_ complexes have been deposited in the Electron Microscopy Data Bank under accession codes EMD-33086 and EMD-33085, respectively. The atomic coordinates of the CCL2–CCR2–G_i_ and apo CCR3–G_i_ complexes have been deposited in the Protein Data Bank under accession codes 7XA3 and 7X9Y, respectively. All other data are available upon request to the corresponding authors.

## References

[1] Griffith JW, Sokol CL, Luster AD (2014). Chemokines and chemokine receptors: positioning cells for host defense and immunity. Annu. Rev. Immunol..

[2] Lazennec G, Richmond A (2010). Chemokines and chemokine receptors: new insights into cancer-related inflammation. Trends Mol. Med..

[3] Zhang C (2018). Eosinophil-derived CCL-6 impairs hematopoietic stem cell homeostasis. Cell Res..

[4] Du X (2021). Eosinophil-derived chemokine (hCCL15/23, mCCL6) interacts with CCR1 to promote eosinophilic airway inflammation. Signal Transduct. Target. Ther..

[5] Li F (2021). Eosinophilic inflammation promotes CCL6-dependent metastatic tumor growth. Sci. Adv..

[6] Kufareva I, Gustavsson M, Zheng Y, Stephens BS, Handel TM (2017). What do structures tell us about chemokine receptor function and antagonism?. Annu. Rev. Biophys..

[7] Hughes CE, Nibbs RJB (2018). A guide to chemokines and their receptors. FEBS J..

[8] Murphy PM (2000). International union of pharmacology. XXII. Nomenclature for chemokine receptors. Pharmacol. Rev..

[9] Isaikina P (2021). Structural basis of the activation of the CC chemokine receptor 5 by a chemokine agonist. Sci. Adv..

[10] Liu K (2020). Structural basis of CXC chemokine receptor 2 activation and signalling. Nature.

[11] Wasilko DJ (2020). Structural basis for chemokine receptor CCR6 activation by the endogenous protein ligand CCL20. Nat. Commun..

[12] Zhang H (2021). Structural basis for chemokine recognition and receptor activation of chemokine receptor CCR5. Nat. Commun..

[13] Shao Z (2021). Identification and mechanism of G protein-biased ligands for chemokine receptor CCR1. Nat. Chem. Biol..

[14] Nagarsheth N, Wicha MS, Zou W (2017). Chemokines in the cancer microenvironment and their relevance in cancer immunotherapy. Nat. Rev. Immunol..

[15] Conductier G, Blondeau N, Guyon A, Nahon JL, Rovère C (2010). The role of monocyte chemoattractant protein MCP1/CCL2 in neuroinflammatory diseases. J. Neuroimmunol..

[16] Grozdanovic M (2019). Novel peptide nanoparticle-biased antagonist of CCR3 blocks eosinophil recruitment and airway hyperresponsiveness. J. Allergy Clin. Immunol.

[17] Shen HH, Xu F, Zhang GS, Wang SB, Xu WH (2006). CCR3 monoclonal antibody inhibits airway eosinophilic inflammation and mucus overproduction in a mouse model of asthma. Acta Pharma. Sin..

[18] Ben S (2008). Treatment with anti-CC chemokine receptor 3 monoclonal antibody or dexamethasone inhibits the migration and differentiation of bone marrow CD34 progenitor cells in an allergic mouse model. Allergy.

[19] Edward Zhou X, Melcher K, Eric Xu H (2019). Structural biology of G protein-coupled receptor signaling complexes. Protein Sci..

[20] Weis WI, Kobilka BK (2018). The molecular basis of G protein-coupled receptor activation. Annu. Rev. Biochem..

[21] Gosselin RD (2005). Constitutive expression of CCR2 chemokine receptor and inhibition by MCP-1/CCL2 of GABA-induced currents in spinal cord neurones. J. Neurochem..

[22] Wan Y (2002). Identification of full, partial and inverse CC chemokine receptor 3 agonists using [35S]GTPgammaS binding. Eur. J. Pharmacol..

[23] Pease JE, Horuk R (2014). Recent progress in the development of antagonists to the chemokine receptors CCR3 and CCR4. Expert Opin. Drug Discov..

[24] Miao M, De Clercq E, Li G (2020). Clinical significance of chemokine receptor antagonists. Expert Opin. drug Metab. Toxicol..

[25] Duan J (2020). Cryo-EM structure of an activated VIP1 receptor-G protein complex revealed by a NanoBiT tethering strategy. Nat. Commun..

[26] Zhou F (2020). Structural basis for activation of the growth hormone-releasing hormone receptor. Nat. Commun..

[27] Xia R (2021). Cryo-EM structure of the human histamine H(1) receptor/G(q) complex. Nat. Commun..

[28] Ballesteros, J. A. & Weinstein, H. in *Methods in Neurosciences* Vol. 25 (ed Sealfon, S. C), 366–428 (Academic Press, 1995).

[29] Crump MP (1997). Solution structure and basis for functional activity of stromal cell-derived factor-1; dissociation of CXCR4 activation from binding and inhibition of HIV-1. EMBO J..

[30] Sanchez J, Lane JR, Canals M, Stone MJ (2019). Influence of chemokine N-terminal modification on biased agonism at the chemokine receptor CCR1. Int. J. Mol. Sci..

[31] Zheng Y (2017). Structure of CC chemokine receptor 5 with a potent chemokine antagonist reveals mechanisms of chemokine recognition and molecular mimicry by HIV. Immunity.

[32] Zheng Y (2016). Structure of CC chemokine receptor 2 with orthosteric and allosteric antagonists. Nature.

[33] Kato HE (2019). Conformational transitions of a neurotensin receptor 1-G(i1) complex. Nature.

[34] Xu P (2021). Structural insights into the lipid and ligand regulation of serotonin receptors. Nature.

[35] Zhuang Y (2021). Structural insights into the human D1 and D2 dopamine receptor signaling complexes. Cell.

[36] Flock T (2015). Universal allosteric mechanism for Gα activation by GPCRs. Nature.

[37] Zhuang Y (2021). Mechanism of dopamine binding and allosteric modulation of the human D1 dopamine receptor. Cell Res..

[38] Liu X (2019). Mechanism of β(2)AR regulation by an intracellular positive allosteric modulator. Science.

[39] Mao C (2020). Cryo-EM structures of inactive and active GABA(B) receptor. Cell Res..

[40] Mastronarde DN (2005). Automated electron microscope tomography using robust prediction of specimen movements. J. Struct. Biol..

[41] Zheng SQ (2017). MotionCor2: anisotropic correction of beam-induced motion for improved cryo-electron microscopy. Nat. methods.

[42] Zhang K (2016). Gctf: Real-time CTF determination and correction. J. Struct. Biol..

[43] Scheres SH (2012). RELION: implementation of a Bayesian approach to cryo-EM structure determination. J. Struct. Biol..

[44] Lubkowski J (1997). The structure of MCP-1 in two crystal forms provides a rare example of variable quaternary interactions. Nat. Struct. Biol..

[45] Koehl A (2018). Structure of the µ-opioid receptor-G(i) protein complex. Nature.

[46] Pettersen EF (2004). UCSF Chimera—a visualization system for exploratory research and analysis. J. Computational Chem..

[47] Emsley P, Cowtan K (2004). Coot: model-building tools for molecular graphics. Acta Crystallogr. Sect. D Biol. Crystallogr.

[48] Adams PD (2010). PHENIX: a comprehensive Python-based system for macromolecular structure solution. Acta Crystallogr. Sect. D Biol. Crystallogr.

[49] Pettersen EF (2021). UCSF ChimeraX: structure visualization for researchers, educators, and developers. Protein Sci..

