# Supplementary material for: Molecular insights into ligand recognition and activation of chemokine receptors CCR2 and CCR3
Source: Cell Discov. 2022 May 15;8:44. doi: 10.1038/s41421-022-00403-4 (PMC9108096; doi:10.1038/s41421-022-00403-4)
Supplement: Supplementary file 1 — Supplementary Information [file 41421_2022_403_MOESM1_ESM.pdf]

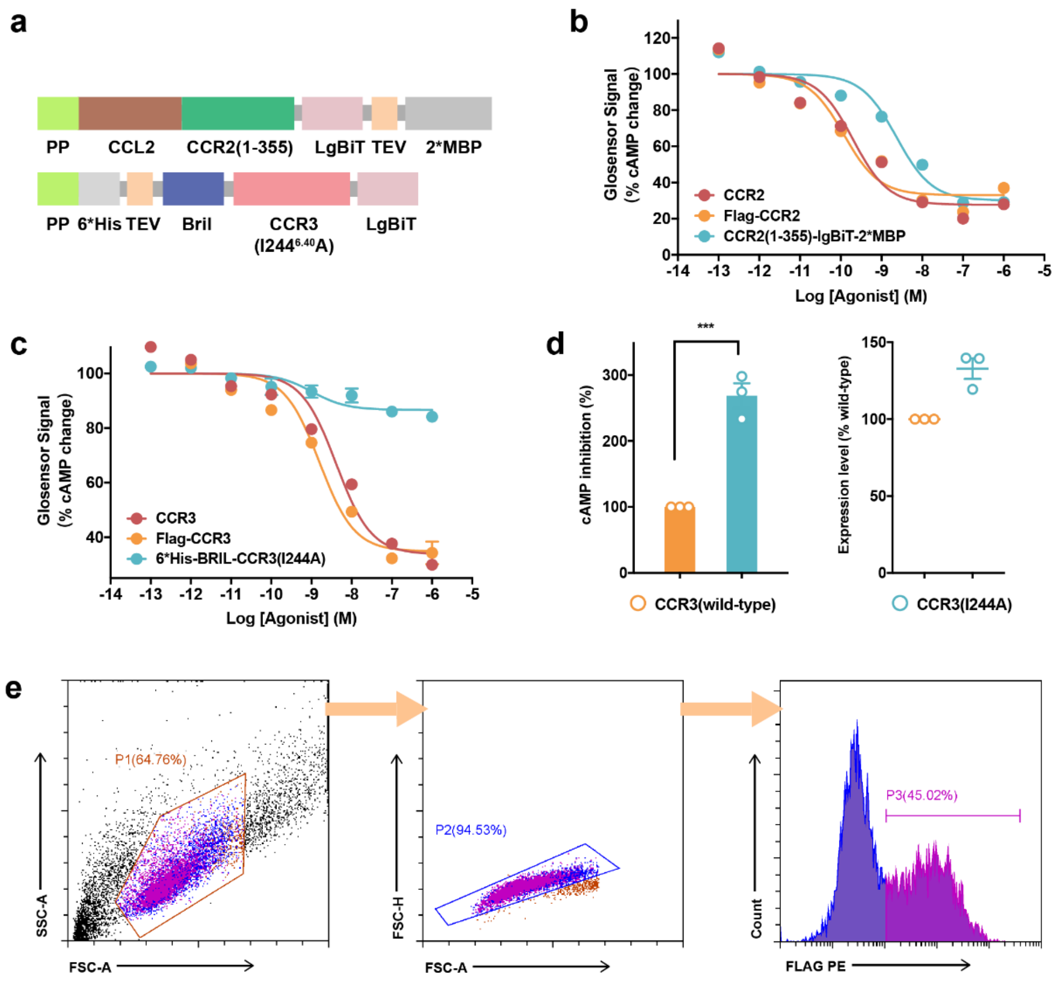

Supplementary Fig. S1: Purification of CCR2– and CCR3–G$_i$ complexes. a Schematic diagrams of CC chemokine receptor-related constructs used in this study (PP: prolactin precursor signal peptide). **b, c** GloSensor cAMP responses for CCR2 (**b**) and CCR3 (**c**) on wild-type and modified receptors, respectively. **d** Effects of CCR3 (I244$^{6.40}$A) mutation on constitutive activity (left) and expression level (right), compared with wild-type CCR3. In **b–d**, *N* = four (**b, c**) or three independent (**d**) experiments, performed with single replicates. P-values were calculated using two-tailed Student's t-test (***P<0.001). All data were shown as mean ± SEM. **e** Representative flow cytometry plots of flow analysis, illustrating the gating strategy for measuring the relative expression of Flag-tagged receptors in HEK 293T cells.

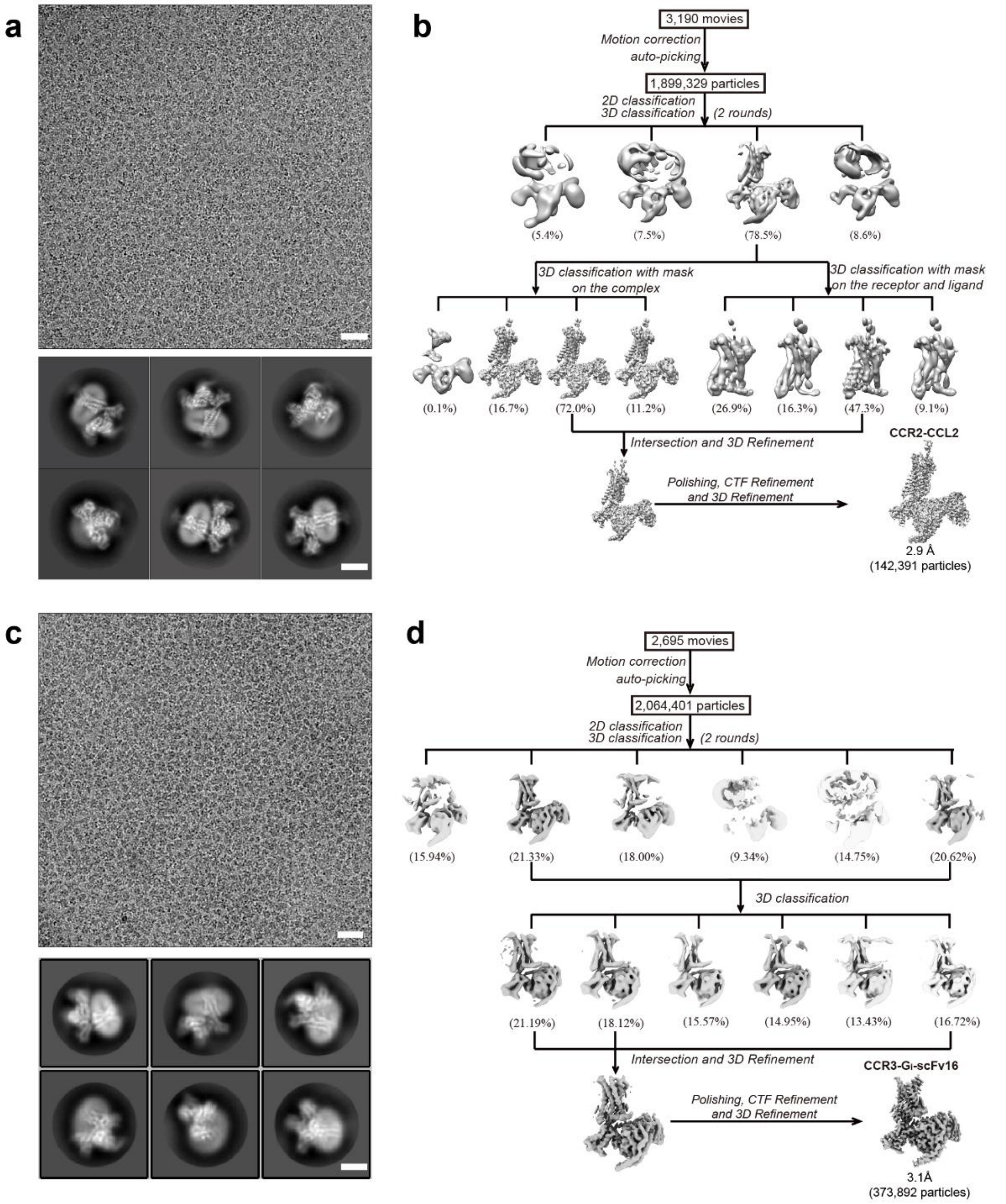

Supplementary Fig. S2: Single particle cryo-EM analysis of CCL2–CCR2–G_i and apo CCR3–G_i complexes. a, c Representative cryo-EM micrographs of CCL2–CCR2–G_i (**a**) and apo CCR3–G_i (**c**) complexes (upper, scale bar: 30 nm). Representative 2D class averages showing distinct secondary structure features from different angles (bottom, scale bar: 5 nm). **b, d** Flowcharts for CCL2–CCR2–G_i (**b**) and apo CCR3-G_i (**d**) complexes.

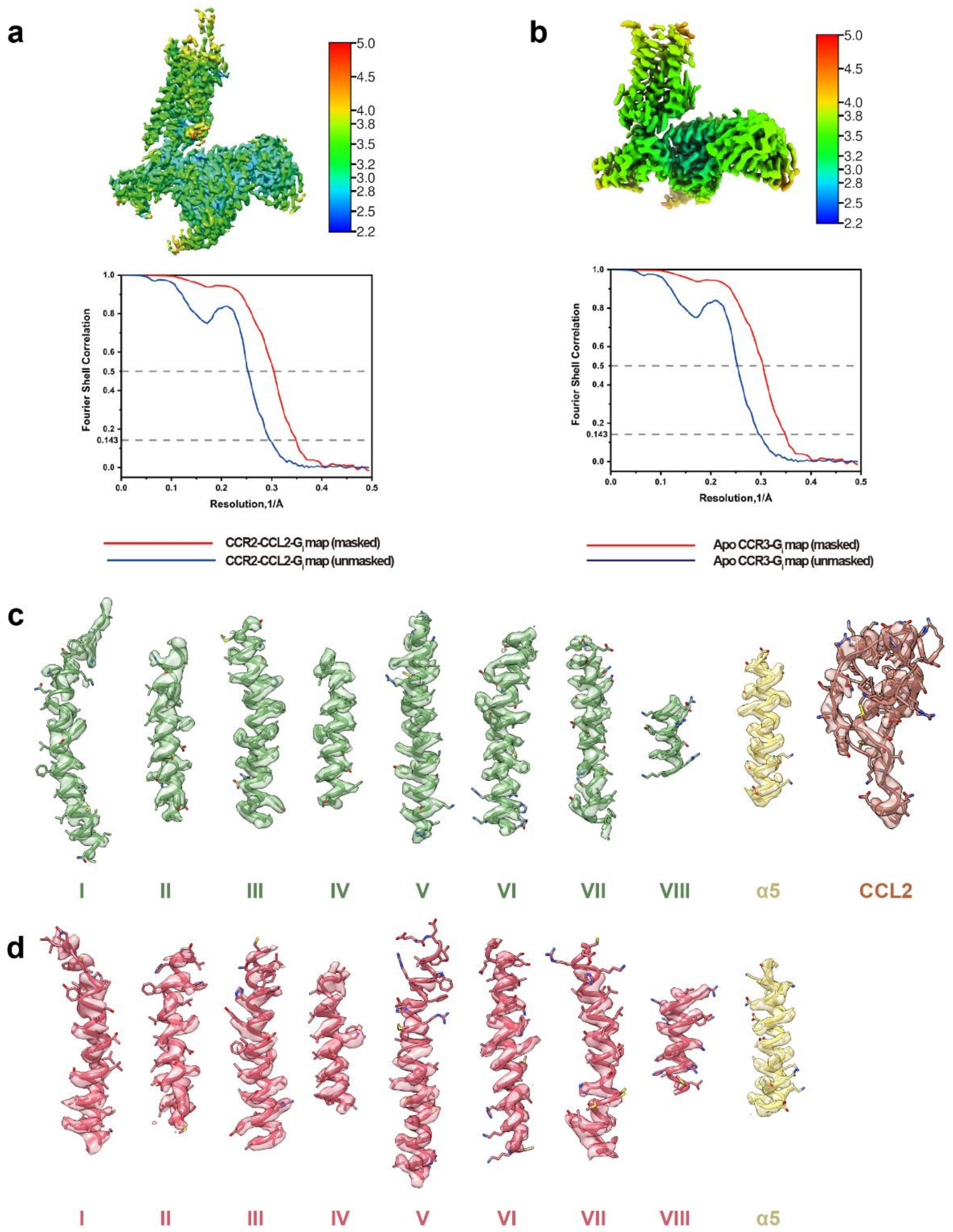

Supplementary Fig. S3: Cryo-EM maps and refined structures. a, b Cryo-EM maps for CCL2–CCR2–G_i (**a**) and apo CCR3–G_i (**b**) complexes, colored by local resolution (Å) calculated using Bsoft package (upper). "Gold-standard" FSC curves (bottom). **c, d** Cryo-EM density maps and models of CCL2–CCR2–G_i (**c**), and apo CCR3–G_i (**d**) complexes were shown for all TM helices, helix 8, CCL2 (**c**), and α-helix of $G\alpha_{i1}$.

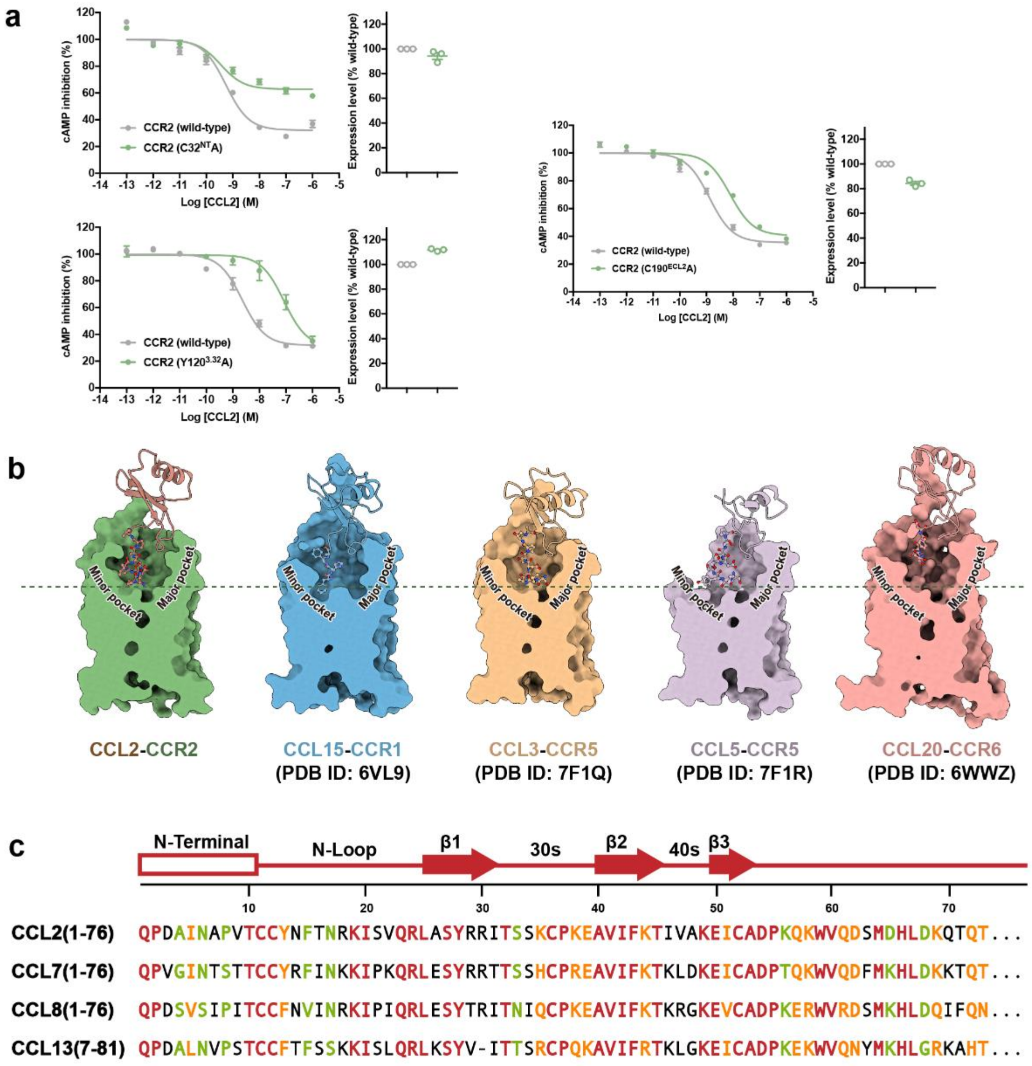

Supplementary Fig. S4: Ligand recognition of CCR2. a Dose-response curves for CCL2-induced G_i signaling on wild-type (grey) and single-site CCR2 mutations (green), measured by Glosensor assay. Data were shown as mean ± SEM; N = three independent experiments, performed with single replicates. **b** Surface cut-away views comparing the binding pockets of chemokine receptors accommodating respective chemokine ligands. Receptors were shown as surface, and chemokines were shown as cartoon with the N-terminus shown as ball and sticks. CCL15–CCR1 (PDB ID: 7VL9); CCL3–CCR5 (PDB ID: 7F1Q); CCL5–CCR5 (PDB ID: 7F1R); CCL20–CCR6 (PDB ID: 6WWZ). **c** Sequence alignment of four human endogenous CCR2 agonists. The positions which shared fully conserved residues were highlighted in red. Residues with strongly similar properties (scoring > 0.5 in the Gonnet PAM 250 matrix) were highlighted in orange. Residues with weakly similar properties (scoring < 0.5 in the Gonnet PAM 250 matrix) were highlighted in green.

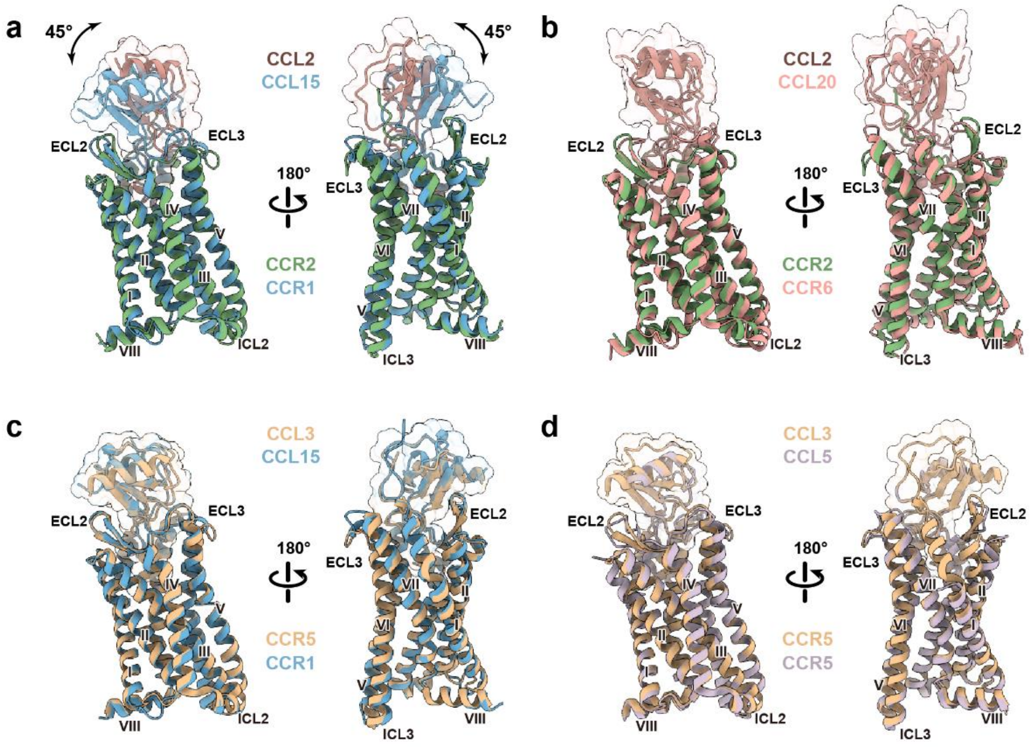

Supplementary Fig. S5: Structural comparison of chemokine–receptor complexes. **a**–**d** Overlay of chemokine-receptors revealed two major orientations of chemokines relative to the corresponding receptors. Receptors were shown as ribbon; chemokines were shown as transparent surface. CCL2 (brown)–CCR2 (green) complex versus CCL15–CCR1 (blue) complex (**a**); CCL2 (brown)–CCR2 (green) complex versus CCL20–CCR6 (pink) complex (**b**); CCL3–CCR5 (yellow) complex versus CCL15–CCR1 (blue) complex (**c**); CCL3–CCR5 (yellow) complex versus CCL5–CCR5 (purple) complex (**d**). CCL15–CCR1 (PDB ID: 7VL9); CCL3–CCR5 (PDB ID: 7F1Q); CCL5–CCR5 (PDB ID: 7F1R); CCL20–CCR6 (PDB ID: 6WWZ).

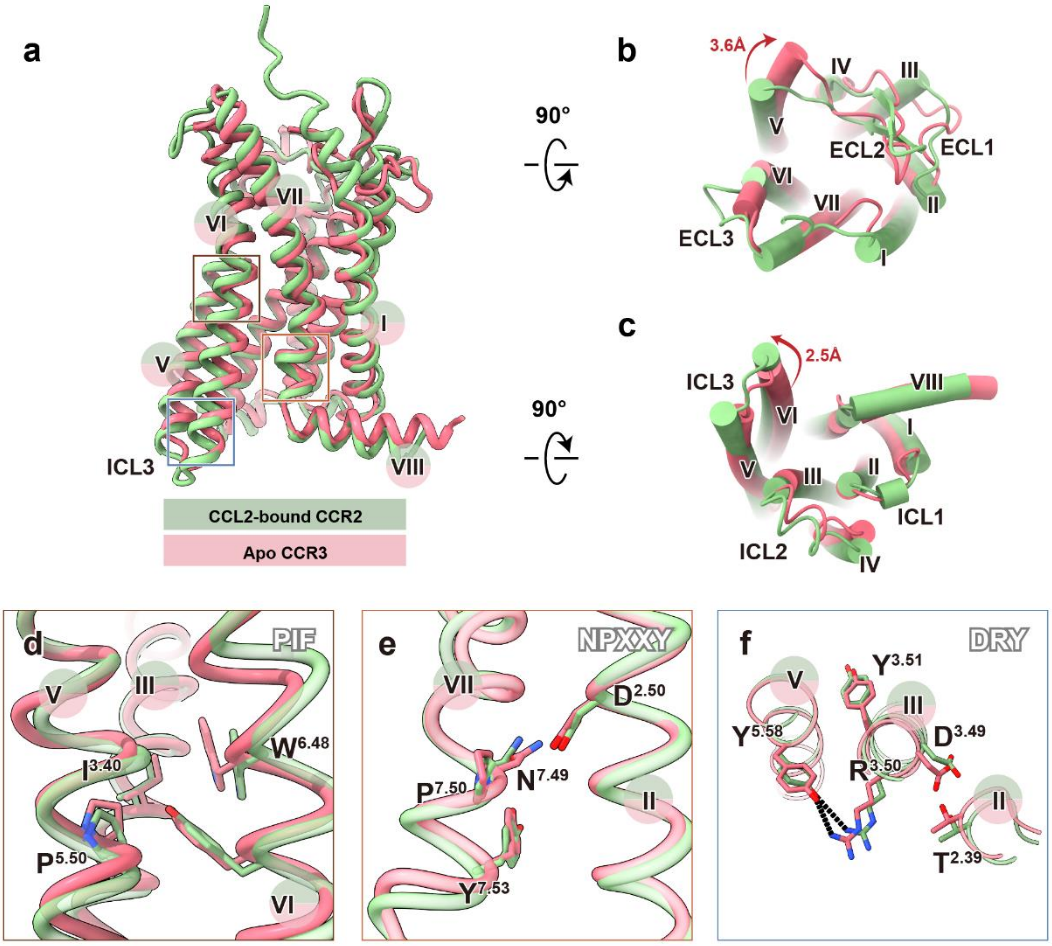

Supplementary Fig. S6: Structural comparison of CCR2 and CCR3 in active states. **a–c** Superimposed structures of apo CCR3 (hot pink) and CCL2-activated CCR2 (green). Side (**a**), extracellular (**b**), and intracellular (**c**) views of the overall structures. **d–f** Three conserved structural motifs of family A receptors labeled according to the amino acid types found in each motif: PIF (**d**), NPXXY (**e**), and DRY (**f**). The coloring scheme was the same as above. Hydrogen bonds were depicted as black dashed lines.

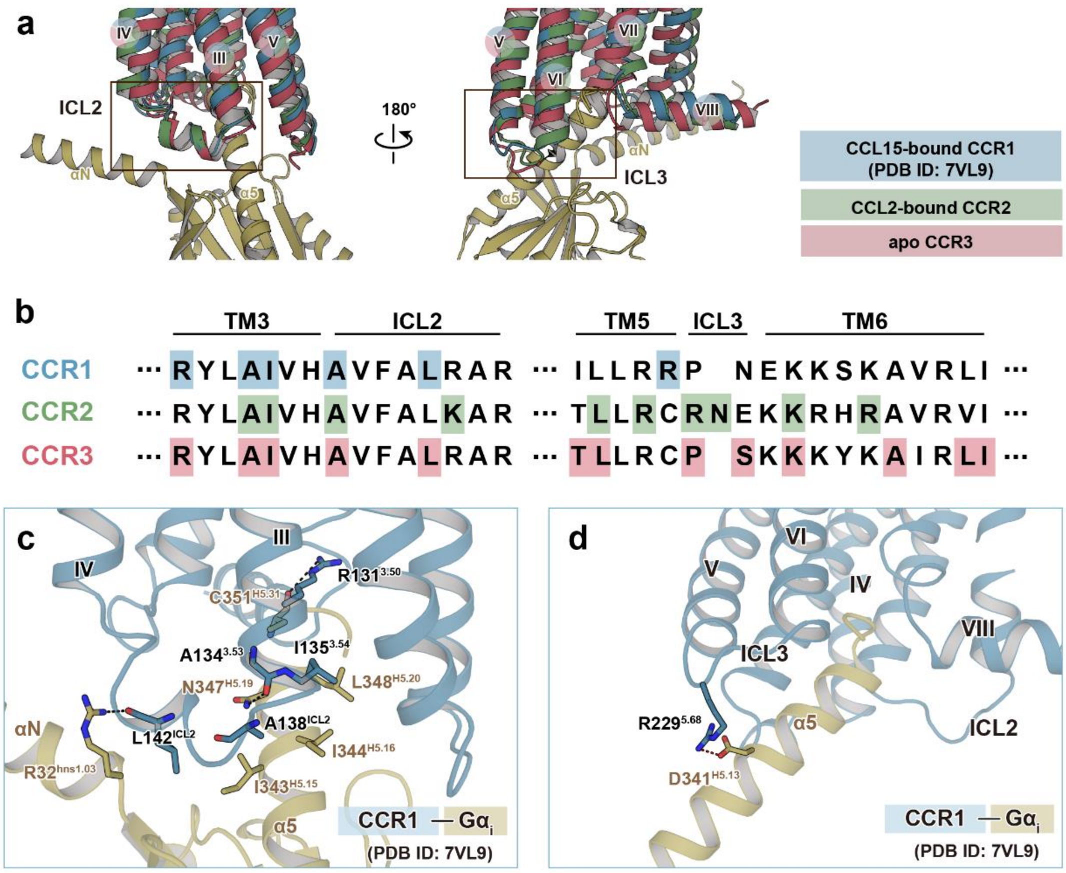

Supplementary Fig. S7: Interactions between chemokine receptors and Gα$_{i1}$. a Comparison of the interface between the α5-helix and cytoplasmic region in CCR1 (blue), CCR2 (green), and CCR3 (pink). The alignment was performed using Gα protein as the reference. **b** Sequence alignment of CCR1, CCR2 and CCR3 in the regions of TM3, ICLs 2–3 and TMs 5–6. The residues which were involved in the interactions with Gα$_i$ were highlighted in blue, green and pink, respectively. **c, d** Detailed interactions between CCR1 (blue) and α5-helix of Gα$_i$ (yellow) around the regions of ICL2 (**c**) and ICL3 (**d**). Hydrogen bonds were depicted as black dashed lines, and electrostatic bonds were depicted as red dashed lines.

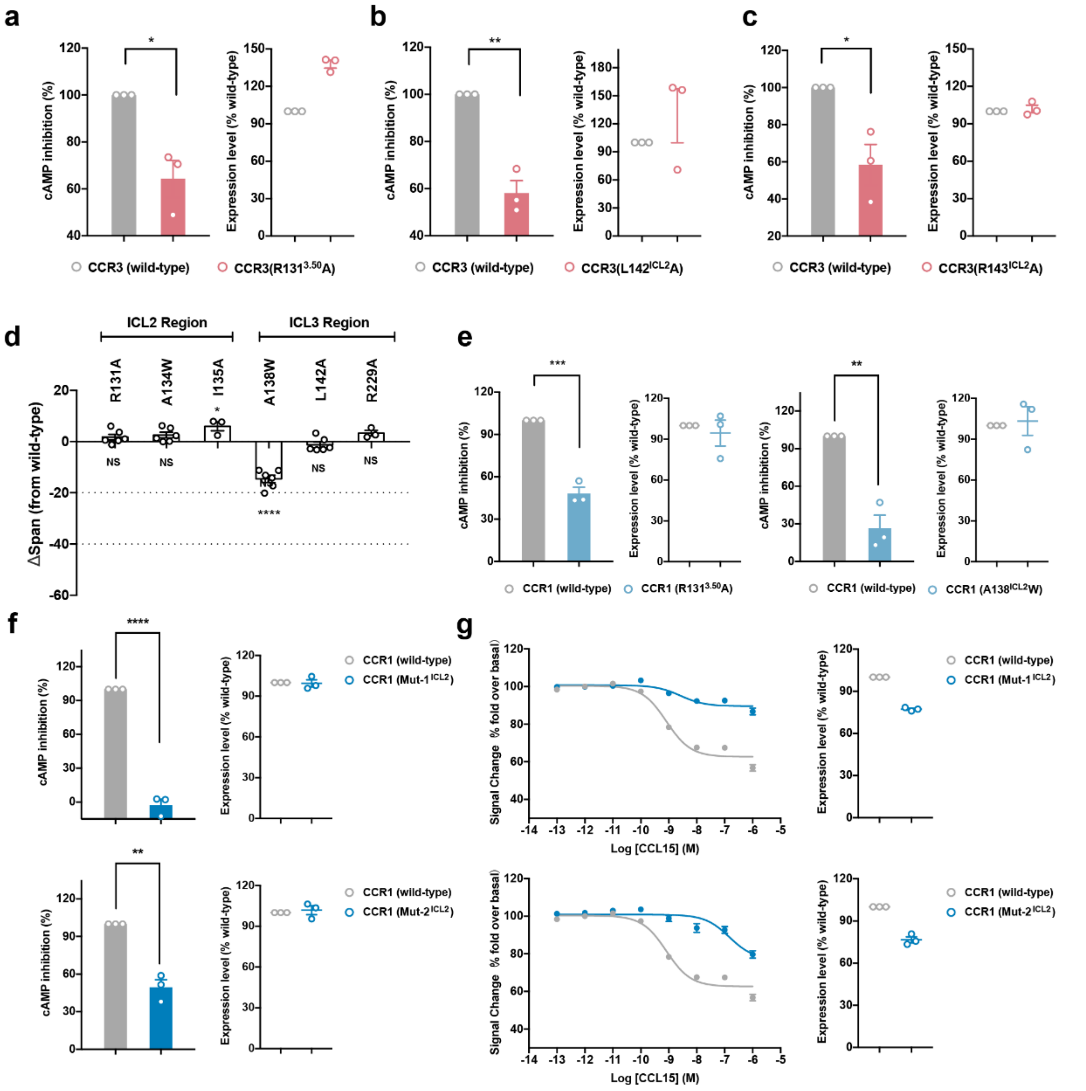

Supplementary Fig. S8: Interactions between CCR1 and Gα_{i1}. a–c Effects of CCR3 (R131$^{3.50}$A, **a**), CCR3 (L142^{ICL2}A, **b**) and CCR3 (R143^{ICL2}A, **c**) on constitutive activities. *N* = three independent experiments, performed with triple replicates. **d** Influence of single-site mutants of CCR1 on agonist-induced cAMP accumulation. *N*= at least three independent experiments performed with single replicates. See **Supplementary Table S7** for detailed statistical evaluation. **e, f** Effects of single- (**e**) and multiple- (**f**) sites mutations of CCR1 on constitutive activities. **g** Concentration-response curves for G_i signaling in NanoBiT G-protein dissociation assay of CCR1 (wild-type) and CCR1 mutations. Mut-1 and Mut-2 represent mutations of CCR1: (R131$^{3.50}$A, A134$^{3.53}$W, A138^{ICL2}W, L142^{ICL2}A), and (R131$^{3.50}$A, A134$^{3.53}$W, A138^{ICL2}W), respectively. In **e–g**, *N* = three independent experiments, performed with quadruple replicates. The difference in fold-change signals between cells transfected with the empty vector plasmid and those with the wild-type receptor reflected the constitutive activity of wild-type CCR3 (**a–c**) or CCR1 (**e–f**), and was set as 100%. The statistical difference between wild-type and mutated receptor was calculated by one-way ANOVA followed by Dunnett's multiple comparisons test (**d**) or two-tailed Student's t-test (**a–c**, **e–f**). Superscripts indicated statistically significant difference

(*P<0.01, **P <0.01, ***P<0.001, ****P<0.0001, and NS was no significance). All data were shown as mean ± SEM.

Supplementary Table S1. Cryo-EM data collection, model refinement, and validation statistics.

	CCL2–CCR2–G_i	Apo CCR3–G_i
**Data collection and processing**		
Magnification	49,310	46,296
Voltage (kV)	300	300
Electron exposure ($e^{-}/Å^2$)	62	65
Defocus range (μm)	-0.5 ~ -2.5	-1.2 ~ -2.2
Pixel size (Å)	1.014	1.08
Symmetry imposed	C1	C1
Initial particle projections (no.)	1,899,329	2,064,401
Final particle projections (no.)	142,391	373,892
Map resolution (Å)	2.9	3.1
FSC threshold	0.143	0.143
Map resolution range (Å)	2.5-5.0	2.5-5.0
**Refinement**		
Initial model used	5T1A	6OS9
Model resolution (Å)	2.9	3.3
FSC threshold	0.5	0.5
Map sharpening *B* factor ($Å^2$)	-98	-118.34
Model composition		
Non-hydrogen atoms	9622	8975
Protein residues	1225	1136
Lipid	0	0
Water	0	0
B factors ($Å^2$)		
Protein	62.93	65.63
Lipids	\	\
R.m.s. deviations		
Bond lengths (Å)	0.006	0.031
Bond angles (°)	1.127	1.978
Validation		
MolProbity score	1.29	1.44
Clashscore	4.07	6.77
Rotamer outliers (%)	0	0.00
Ramachandran plot		
Favored (%)	97.52	97.68
Allowed (%)	2.48	2.32
Disallowed (%)	0	0

Supplementary Table S2. Interactions of CCL2 with CCR2. Residues within 4Å are shown.

CCL2	CCR2	Interaction	Distance (Å)
GLN1	TRP98$^{2.60}$	Hydrophobic interaction	
	CYS113$^{3.25}$	Sidechain-sidechain hydrogen bond	3.86
	THR117$^{3.29}$	Sidechain-sidechain hydrogen bond	2.62
	TYR120$^{3.32}$	Sidechain-backbone hydrogen bond	3.98
	HIS121$^{3.33}$	Sidechain-sidechain hydrogen bond	3.20
	CYS190^{ECL2}	Sidechain-sidechain hydrogen bond	2.84
	GLY191^{ECL2}	Backbone-sidechain hydrogen bond	3.81
	PRO192^{ECL2}	Backbone-sidechain hydrogen bond	3.87
ASP3	GLU291$^{7.39}$	Sidechain-backbone hydrogen bond	3.95
ALA4	LYS38$^{1.28}$	Sidechain-backbone hydrogen bond	3.75
ILE5	LYS38$^{1.28}$	Sidechain-backbone hydrogen bond	3.54
	TRP98$^{2.60}$	Hydrophobic interaction	
PRO8	HIS33^{NT}	Sidechain-backbone hydrogen bond	2.89
VAL9		Sidechain-backbone hydrogen bond	3.69
TYR13	CYS32^{NT}	Hydrophobic interaction	
ARG29	ASP185^{ECL2}	Electrostatic interaction	
SER34	PHE194^{ECL2}	Backbone-sidechain hydrogen bond	3.20
	ASN199$^{5.35}$	Sidechain-backbone hydrogen bond	2.35
CYS36	ARG196$^{5.32}$	Sidechain-backbone hydrogen bond	3.3
PRO37		Sidechain-backbone hydrogen bond	2.93
GLU50	GLY29^{NT}	Backbone-backbone hydrogen bond	3.88
	ALA30^{NT}	Backbone-backbone hydrogen bond	3.16

Supplementary Table S3. Interactions of Gα_i with CCR2. Residues within 4Å are shown.

CCR2	G_i	Interaction	Distance (Å)
ALA141$^{3.53}$	ASN347$^{H5.19}$	Backbone-sidechain hydrogen bond	3.52
ILE142$^{3.54}$	ILE344$^{H5.16}$<br>LEU348$^{H5.20}$<br>LEU353$^{H5.25}$	Hydrophobic interaction	
ALA145^{ICL2}	ILE343$^{H5.15}$<br>ILE344$^{H5.16}$	Hydrophobic interaction	
LYS150^{ICL2}	ASP193$^{s2s3.02}$	Electrostatic interaction	
LEU229$^{5.65}$	LEU348$^{H5.20}$	Hydrophobic interaction	
ARG231$^{5.67}$	ASP341$^{H5.13}$	Electrostatic interaction	
ARG233^{ICL3}	GLU318$^{h4s6.12}$	Electrostatic interaction	
ASN234^{ICL3}	ASP315$^{h4s6.09}$	Sidechain-backbone hydrogen bond	3.34
LYS237$^{6.29}$	PHE354$^{H5.26}$	Hydrophobic interaction	
ARG240$^{6.32}$	PHE354$^{H5.26}$	Sidechain-backbone hydrogen bond	2.75
GLY309^{H8}	GLY352$^{H5.24}$	Sidechain-backbone hydrogen bond	3.63
GLU310^{H8}	PHE354$^{H5.26}$	Backbone-backbone hydrogen bond	3.44
LYS311^{H8}	ASP350$^{H5.22}$	Electrostatic interaction	

Supplementary Table S4. Interactions of Gα_i with CCR3. Residues within 4Å are shown.

CCR3	G_i	Interaction	Distance (Å)
ARG63^{ICL1}	ASP350$^{H5.22}$	Sidechain-sidechain hydrogen bond	3.49
ARG66^{ICL1}	GLU28$^{HN.52}$	Sidechain-sidechain hydrogen bond	2.98
THR69$^{2.39}$	CYS351$^{H5.23}$	Sidechain-sidechain hydrogen bond	3.76
ASN70$^{2.40}$	ASP350$^{H5.22}$	Sidechain-backbone hydrogen bond	3.15
	CYS351$^{H5.23}$	Sidechain-backbone hydrogen bond	2.64
ARG131$^{3.50}$	GLY352$^{H5.24}$	Backbone-sidechain hydrogen bond	3.93
	LEU353$^{H5.25}$	Hydrophobic interaction	
ALA134$^{3.53}$	ASN347$^{H5.19}$	Backbone-sidechain hydrogen bond	2.75
ILE135$^{3.54}$	LEU344$^{H5.16}$	Hydrophobic interaction	
	LEU348$^{H5.20}$	Hydrophobic interaction	
ALA138^{ICL2}	ILE343$^{H5.15}$	Hydrophobic interaction	
	ILE344$^{H5.16}$	Hydrophobic interaction	
VAL139^{ICL2}	LEU194$^{S3.01}$	Hydrophobic interaction	
	PHE336$^{H5.08}$	Hydrophobic interaction	
LEU142^{ICL2}	LEU194$^{S3.01}$	Hydrophobic interaction	
	ILE343$^{H5.15}$	Hydrophobic interaction	
ARG143^{ICL2}	ASP193$^{s2s3.02}$	Electrostatic interaction	
THR225$^{5.64}$<br>LEU226$^{5.65}$	ILE344$^{H5.15}$	Hydrophobic interaction	
PRO230^{ICL3}	TYR320$^{S6.02}$	Hydrophobic interaction	
SER231^{ICL3}	GLU318$^{h4s6.12}$	Sidechain-sidechain hydrogen bond	2.64
LYS233$^{6.29}$	PHE354$^{H5.26}$	Hydrophobic interaction	
ALA237$^{6.33}$	ILE348$^{H5.20}$	Hydrophobic interaction	
	LEU353$^{H5.25}$	Backbone-backbone hydrogen bond	3.47
LEU240$^{6.36}$<br>ILE241$^{6.37}$	LEU353$^{H5.25}$	Hydrophobic interaction	
GLU306^{H8}	PHE354$^{H5.26}$	Backbone-backbone hydrogen bond	2.71
ARG307^{H8}	ASN346$^{H5.18}$	Sidechain-sidechain hydrogen bond	3.67
ARG307^{H8}	LYS349$^{H5.21}$	Backbone-backbone hydrogen bond	3.22
ARG307^{H8}	ASP350$^{H5.22}$	Sidechain-sidechain hydrogen bond	3.38

Supplementary Table S5. Effects of single-site mutations on CCR2.

	pEC_{50}±SEM	Span±SEM	N	Expression (%wild-type)
Wild-type	9.23±0.26	69.7±1.2	9	100.0±0.0
Mutations around ICL2				
$A141^{3.53}W$	8.23±0.18	46.5±4.7****	3	90.1±2.3
$I142^{3.54}A$	8.89±0.24	72.1±1.5	3	147.5±2.2
$A145^{ICL2}W$	8.89±0.24	31.9±2.6****	3	48.2±2.9
$K150^{ICL2}A$	9.50±0.22	66.6±0.4	3	111.4±2.0
Mutations around ICL3				
$L229^{5.68}A$	9.72±0.24	72.0±2.9	3	100.2±1.7
$R231^{5.67}A$	9.81±0.09	67.4±0.4	3	75.1±3.1
$R233^{ICL3}A$	9.64±0.42	71.3±1.1	3	85.0±1.9
$N234^{ICL3}A$	9.29±0.15	68.5±2.1	3	149.9±6.0
$K237^{6.29}A$	9.50±0.21	68.5±1.7	3	190.5±0.7
$R240^{6.32}A$	9.17±0.20	75.3±2.2	3	66.8±2.9

Glosensor results of cAMP accumulation for wild-type and mutated CCR2. The span was defined as the window between the response induced by agonist at maximum (E_{max}) and minimum concentration. *N*= at least three independent experiments performed with single replicates as shown. The statistical difference between wild-type and mutated CCR2 was calculated by one-way ANOVA followed by Dunnett's multiple comparisons test. Superscripts indicated statistically significant difference (****P<0.0001). All data were shown as mean ± SEM.

Supplementary Table S6. Effects of single-site mutations on CCR3.

	pEC_{50}±SEM	Span±SEM	N	Expression (%wild-type)
Wild-type	8.54±0.12	75.0±1.3	25	100.0±0.0
Mutations around ICL2				
R131$^{3.50}$A	8.80±0.45	27.4±5.4****	5	152.9±17.0
A134$^{3.53}$W	8.49±0.30	43.4±1.8****	3	23.6±0.4
I135$^{3.54}$A	7.64±0.35	54.8±4.7***	3	37.3±0.9
A138^{ICL2}W	8.81±1.04	33.8±3.4****	3	13.7±1.3
L142^{ICL2}A	8.44±0.30	68.8±3.4	5	145.8±3.3
R143^{ICL2}A	8.26±0.07	74.6±1.2	6	48.0±6.4
Mutations around ICL3				
T225$^{5.64}$A	8.67±0.18	72.4±1.1	5	104.0±1.5
L226$^{5.65}$A	8.12±0.32	51.4±3.5****	5	89.8±2.0
P230^{ICL3}A	8.48±0.20	66.7±1.4	3	25.5±1.5
S231^{ICL3}A	8.07±0.22	70.0±0.6	6	59.3±3.7
K233$^{6.29}$A	8.41±0.11	72.1±1.4	5	130.4±2.1
A237$^{6.33}$W	7.75±0.17	62.0±4.0**	5	39.7±1.9
L240$^{6.36}$A	8.20±0.31	60.0±2.1**	4	119.0±2.7
I241$^{6.37}$A	8.46±0.13	78.1±2.4	6	119.1±4.5

Glosensor results of cAMP accumulation for wild-type and mutated CCR3. The span was defined as the window between the response induced by agonist at maximum (E_{max}) and minimum concentration. *N*= at least three independent experiments performed with single replicates. The statistical difference between wild-type and mutated CCR3 was calculated by one-way ANOVA followed by Dunnett's multiple comparisons test. Superscripts indicated statistically significant difference (**$P<0.01$, ***$P<0.001$, and ****$P<0.0001$). All data were shown as mean ± SEM.

Supplementary Table S7. Effects of single-site mutations on CCR1.

	pEC_{50}±SEM	Span±SEM	N	Expression (%wild-type)
Wild-type	8.98±0.08	39.9±0.9	16	100.0±0.0
Mutations around ICL2				
$R131^{3.50}A$	9.14±0.35	39.4±1.4	6	91.4±2.5
$A134^{3.53}W$	9.30±0.20	40.3±1.8	6	102.9±3.4
$I135^{3.54}A$	8.88±0.12	48.2±1.7**	3	82.6±4.0
$A138^{ICL2}W$	9.62±0.08*	25.9±2.5****	6	98.1±5.3
$L142^{ICL2}A$	9.38±0.19	36.6±1.3	6	101.1±2.4
Mutations around ICL3				
$R229^{5.68}A$	9.02±0.08	45.7±0.4	3	100.2±1.8

NanoBiT results of G-protein dissociation for wild-type and mutated CCR1. The span was defined as the window between the response induced by agonist at maximum (E_{max}) and minimum concentration. *N*= at least three independent experiments performed with single replicates. The statistical difference between wild-type and mutated CCR1 was calculated by one-way ANOVA followed by Dunnett's multiple comparisons test. Superscripts indicated statistically significant difference (**P<0.01 and ****P<0.0001). All data were shown as mean ± SEM.

Supplementary Information: Amino acid sequences of the constructs involved in complex formation and purification.

CCL2-CCR2-LgBiT-2*MBP

Coloring denotes the prolactin precursor (pp) signal peptide (orange), CCL2(purple), the flexible linker (green), CCR2 (black), TEV protease cleavage site (yellow), LgBiT (red) and double maltose-binding protein (grey).

MDSKGSSQKGSRLLLLLVVSNLLLCQGVVSQPDAINAPVTCCYNFTNRKISVQRLAS
YRRITSSKCPKEAVIFKTIVAKEICADPKQKWVQDSMDHLDKQTQTPKTMLSTSRSR
FIRNTNESGEEVTTFFDYDYGAPCHKFDVKQIGAQLLPPLYSLVFIFGFVGNMLVVL
ILINCKKLKCLTDIYLLNLAISDLLFLITLPLWAHSAANEWVFGNAMCKLFTGLYHI
GYFGGIFFIILLTIDRYLAIVHAVFALKARTVTFGVVTSVITWLVAVFASVPGIIFT
KCQKEDSVYVCGPYFPRGWNNFHTIMRNILGLVLPLLIMVICYSGILKTLLRCRNEK
KRHRAVRVIFTIMIVYFLFWTPYNIVILLNTFQEFFGLSNCESTSQLDQATQVTETL
GMTHCCINPIIYAFVGEKFRRYLSVFFRKHITKRFCKQCPVFVFTLEDFVGDWEQTA
AYNLDQVLEQGGVSSLLQNLAVSVTPIQRIVRSGENALKIDIHVIIPYEGLSADQMA
QIEEVFKVVYPVDDHHFKVILPYGTLVIDGVTPNMLNYFGRPYEGIAVFDGKKITVT
GTLWNGNKIIDERLITPDGSMLFRVTINSGGSENLYFQGAKIEEGKLVIWINGDKGY
NGLAEVGKKFEKDTGIKVTVEHPDKLEEKFPQVAATGDGPDIIFWAHDRFGGYAQSG
LLAEITPDKAFQDKLYPFTWDAVRYNGKLIAYPIAVEALSLIYNKDLLPNPPKTWEE
IPALDKELKAKGKSALMFNLQEPYFTWPLIAADGGYAFKYENGKYDIKDVGVDNAGA
KAGLTFLVDLIKNKHMNADTDYSIAEAAFNKGETAMTINGPWAWSNIDTSKVNYGVT
VLPTFKGQPSKPFVGVLSAGINAASPNKELAKEFLENYLLTDEGLEAVNKDKPLGAV
ALKSYEEELAKDPRIAATMENAQKGEIMPNIPQMSAFWYAVRTAVINAASGRQTVDE
ALKDAQTAKIEEGKLVIWINGDKGYNGLAEVGKKFEKDTGIKVTVEHPDKLEEKFPQ
VAATGDGPDIIFWAHDRFGGYAQSGLLAEITPDKAFQDKLYPFTWDAVRYNGKLIAY
PIAVEALSLIYNKDLLPNPPKTWEEIPALDKELKAKGKSALMFNLQEPYFTWPLIAA
DGGYAFKYENGKYDIKDVGVDNAGAKAGLTFLVDLIKNKHMNADTDYSIAEAAFNKG
ETAMTINGPWAWSNIDTSKVNYGVTVLPTFKGQPSKPFVGVLSAGINAASPNKELAK
EFLENYLLTDEGLEAVNKDKPLGAVALKSYEEELAKDPRIAATMENAQKGEIMPNIP
QMSAFWYAVRTAVINAASGRQTVDEALKDAQTRITK

BRIL-CCR3 (I244A)-LgBiT

Coloring denotes prolactin precursor (pp) signal peptide (orange), BRIL fusion protein (purple), the flexible linker (green), CCR3 (black, the I244A mutation is highlighted in blue), TEV protease cleavage site (yellow), LgBiT (red) and His tag (pink).

MDSKGSSQKGSRLLLLLVVSNLLLCQGVVSHHHHHHSSGLVPRGSHMASHHHHHHHH
HHGSAENLYFQGADLEDNWETLNDNLKVIEKADNAAQVKDALTKMRAAALDAQKATP
PKLEDKSPDSPEMKDFRHGFDILVGQIDDALKLANEGKVKEAQAAAEQLKTTRNAYI
QKYLMTTSLDTVETFGTTSYYDDVGLLCEKADTRALMAQFVPPLYSLVFTVGLLGNV
VVVMILIKYRRLRIMTNIYLLNLAISDLLFLVTLPFWIHYVRGHNWVFGHGMCKLLS
GFYHTGLYSEIFFIILLTIDRYLAIVHAVFALRARTVTFGVITSIVTWGLAVLAALP
EFIFYETEELFEETLCSALYPEDTVYSWRHFHTLRMTIFCLVLPLLVMAICYTGIIK

TLLRCPSKKKYKAIRLIFVAMAVFFIFWTPYNVAILLSSYQSILFGNDCERSKHLDL
VMLVTEVIAYSHCCMNPVIYAFVGERFRKYLRHFFHRHLLMHLGRYIPFLPSEKLER
TSSVSPSTAEPELSIVFGGSSGVFTLEDFVGDWEQTAAYNLDQVLEQGGVSSLLQNL
AVSVTPIQRIVRSGENALKIDIHVIIPYEGLSADQMAQIEEVFKVVYPVDDHHFKVI
LPYGTLVIDGVTPNMLNYFGRPYEGIAVFDGKKITVTGTLWNGNKIIDERLITPDGS
MLFRVTINS

CCR2-bound DNGα_{i1}

The mutations are highlighted in red.

MGCTLSAEDKAAVERSKMIDRNLREDGEKAAREVKLLLLGAGESGKSTIVKQMKIIT
EAGYSEEECKQYKAVVYSNTIQSIIAIIRAMGRLKIDFGDSARADDARQLFVLAGAA
EEGFMTAELAGVIKRLWKDSGVQACFNRSREYQLNDSAAYYLNDLDRIAQPNYIPTQ
QDVLRTRVKTTGIVETHFTFKDLHFKMFDVGAQRSERKKWIHCFEGVTAIIFCVALS
DYDLVLAEDEEMNRMHESMKLFDSICNNKWFTDTSIILFLNKKDLFEEKIKKSPLTI
CYPEYAGSNTYEEAAAYIQCQFEDLNKRKDTKEIYTHFTCSTDTKNVQFVFDAVTDV
IIKNNLKDCGLF

CCR3-bound DNGα_{i1}

The mutations are highlighted in red.

MGCTLSAEDKAAVERSKMIDRNLREDGEKAAREVKLLLLGAGESGKNTIVKQMKIIT
EAGYSEEECKQYKAVVYSNTIQSIIAIIRAMGRLKIDFGDSARADDARQLFVLAGAA
EEGFMTAELAGVIKRLWKDSGVQACFNRSREYQLNDSAAYYLNDLDRIAQPNYIPTQ
QDVLRTRVKTTGIVETHFTFKDLHFKMFDVGAQRSERKKWIHCFEGVTAIIFCVALS
DYDLVLAEDEEMNRMHASMKLFDSICNNKWFTDTSIILFLNKKDLFEEKIKKSPLTI
CYPEYAGSNTYEEAAAYIQCQFEDLNKRKDTKEIYTHFTCSTDTKNVQFVFDAVTDV
IIKNNLKDCGLF

Gβ1-peptide 86

Coloring denotes the flexible linker (green), Gβ (black), 3C protease cleavage site (blue), peptide 86 (yellow) and His tag (pink).

MHHHHHHLEVLFQGPGSSGSELDQLRQEAEQLKNQIRDARKACADATLSQITNNIDP
VGRIQMRTRRTLRGHLAKIYAMHWGTDSRLLVSASQDGKLIIWDSYTTNKVHAIPLR
SSWVMTCAYAPSGNYVACGGLDNICSIYNLKTREGNVRVSRELAGHTGYLSCCRFLD
DNQIVTSSGDTTCALWDIETGQQTTTFTGHTGDVMSLSLAPDTRLFVSGACDASAKL
WDVREGMCRQTFTGHESDINAICFFPNGNAFATGSDDATCRLFDLRADQELMTYSHD
NIICGITSVSFSKSGRLLLAGYDDFNCNVWDALKADRAGVLAGHDNRVSCLGVTDDG
MAVATGSWDSFLKIWNGSSGGGGSGGGGSSGVSGWRLFKKIS

Gγ2

MASNNTASIAQARKLVEQLKMEANIDRIKVSKAAADLMAYCEAHAKEDPLLTPVPAS
ENPFREKKFFCAIL
